# RAD52 and ERCC6L/PICH have a compensatory relationship for genome stability in mitosis

**DOI:** 10.1371/journal.pgen.1011479

**Published:** 2024-11-19

**Authors:** Beth Osia, Arianna Merkell, Felicia Wednesday Lopezcolorado, Xiaoli Ping, Jeremy M. Stark

**Affiliations:** Department of Cancer Genetics and Epigenetics, Beckman Research Institute of the City of Hope, Duarte, California, United States of America; Columbia University, UNITED STATES OF AMERICA

## Abstract

Mammalian RAD52 is a DNA repair factor with strand annealing and recombination mediator activities that appear important in both interphase and mitotic cells. Nonetheless, RAD52 is dispensable for cell viability. To query RAD52 synthetic lethal relationships, we performed genome-wide CRISPR knock-out screens and identified hundreds of candidate synthetic lethal interactions. We then performed secondary screening and identified genes for which depletion causes reduced viability and elevated genome instability (increased 53BP1 nuclear foci) in RAD52-deficient cells. One such factor was ERCC6L, which marks DNA bridges during anaphase, and hence is important for genome stability in mitosis. Thus, we investigated the functional interrelationship between RAD52 and ERCC6L. We found that RAD52 deficiency increases ERCC6L-coated anaphase ultrafine bridges, and that ERCC6L depletion causes elevated RAD52 foci in prometaphase and interphase cells. These effects were enhanced with replication stress (i.e. hydroxyurea) and topoisomerase IIα inhibition (ICRF-193), where post-treatment effect timings were consistent with defects in addressing stress in mitosis. Altogether, we suggest that RAD52 and ERCC6L co-compensate to protect genome stability in mitosis.

## Author summary

Mammalian RAD52 is a protein that protects DNA integrity through its roles in DNA repair and replication, and is being developed as a target for cancer treatment. To identify factors that influence dependence on RAD52 to maintain cell viability, we performed screens to identify genes that negatively impact cell viability and increase genome instability when disrupted in cells that also lack RAD52. Through these screens, we identified ERCC6L/PICH, a protein that helps resolve ultrafine DNA bridges, which can disrupt the segregation of sister chromatids during anaphase of mitosis. In characterizing the relationship between ERCC6L and RAD52, we found that RAD52 loss increases the number of DNA bridges marked by ERCC6L. Likewise, we discovered that ERCC6L depletion causes RAD52 to accumulate into foci in both mitosis and interphase. These effects were enhanced when loss of each protein was combined with agents that induce additional genotoxic stress (e.g., DNA replication stress). Our findings point to a model where RAD52 compensates for genotoxic stress caused by ERCC6L loss, and *vice versa*.

## Introduction

Human RAD52 (radiation sensitive 52) is a protein that self-associates to form an oligomeric ring structure and exhibits DNA-binding activities for both double-stranded and single-stranded DNA, which enables RAD52 to perform both annealing [1–3] and strand exchange functions [2]. RAD52 is also important for several types of homology-driven DNA repair, including single strand annealing (SSA) [4–7], homologous recombination (HR) associated with transcription [8–12], and mitotic DNA synthesis (MiDAS) [13–18]. The latter (MiDAS) refers to synthesis that occurs in early mitosis (e.g., prophase) to repair under-replicated regions, which appears to be particularly important for replication of common fragile sites [13,16,19,20]. In addition to its roles in these aspects of HR, RAD52 is involved in alternative lengthening of telomeres (ALT) [21,22] and plays a protective role at replication forks by preventing unscheduled fork reversal and degradation [23].

While RAD52 appears to be involved in several aspects of genome maintenance, the circumstances that require such functions of RAD52 remain poorly understood, particularly because RAD52 is not essential for viability [24]. One approach to identifying such circumstances is to evaluate synthetic lethal relationships with RAD52. Synthetic lethality is generally defined as the disruption of two cellular factors resulting in reduction of viability or proliferation, while perturbation of either factor individually does not have such an effect [25,26]. RAD52 has been reported to show synthetic lethal relationships with several HR factors [2,25,27–30]. Beyond such HR deficiencies, the range of synthetic lethal interactions with RAD52 is largely undefined. Given that numerous RAD52 small molecule inhibitors have been described that may be translatable for use in the clinic [25,27,31], expanding the understanding of when cells become reliant on RAD52 will inform the successful application of such inhibitors in cancer treatment.

Here, we describe a set of synthetic lethality screens with RAD52 loss, and from secondary screening, describe a set of studies with ERCC6L (also known as PICH). ERCC6L is a SWI-SNF2-family DNA translocase involved in the resolution of DNA bridges that form between sister chromatids in mitosis due to incomplete replication [32–36]. ERCC6L has also been reported to primarily promote genome stability during mitosis, and is excluded from the nucleus prior to mitotic nuclear envelope breakdown [35,37]. In mitosis, ERCC6L localizes to ultrafine DNA bridges (UFBs), which are DNA bridges that are not readily stained by DNA dyes [36]. Such UFBs form at points of sister chromatid nondisjunction as cells prepare to enter mitotic anaphase [36]. Evidence supports that ERCC6L initiates the dissolution of UFBs in a process involving recruitment of BLM, topoisomerase IIIα (Topo IIIα), RMI1, and RMI2 (together known as the BTRR complex), and topoisomerase IIα (Topo IIα) [35–41]. Biochemical analysis of ERCC6L suggests that the protein recognizes and binds to DNA under tension (e.g., that of DNA catenanes as sister chromatids segregate during anaphase), and that ERCC6L, together with Topo IIIα, produces positive supercoiling required for rapid decatenation of sister chromatids by Topo IIα at anaphase onset [42,43]. Given that RAD52 and ERCC6L are both implicated in genome maintenance in mitosis, and because we identified ERCC6L in our synthetic lethal screen with RAD52, we sought to examine the interplay between these two factors. In particular, we have investigated the influence of RAD52 on formation of ERCC6L-UFBs, and conversely the influence of ERCC6L on localization of RAD52 into foci.

## Results

### Genome-wide CRISPR knockout screen identifies pathways that are synthetic lethal with RAD52

**Fig 1:**
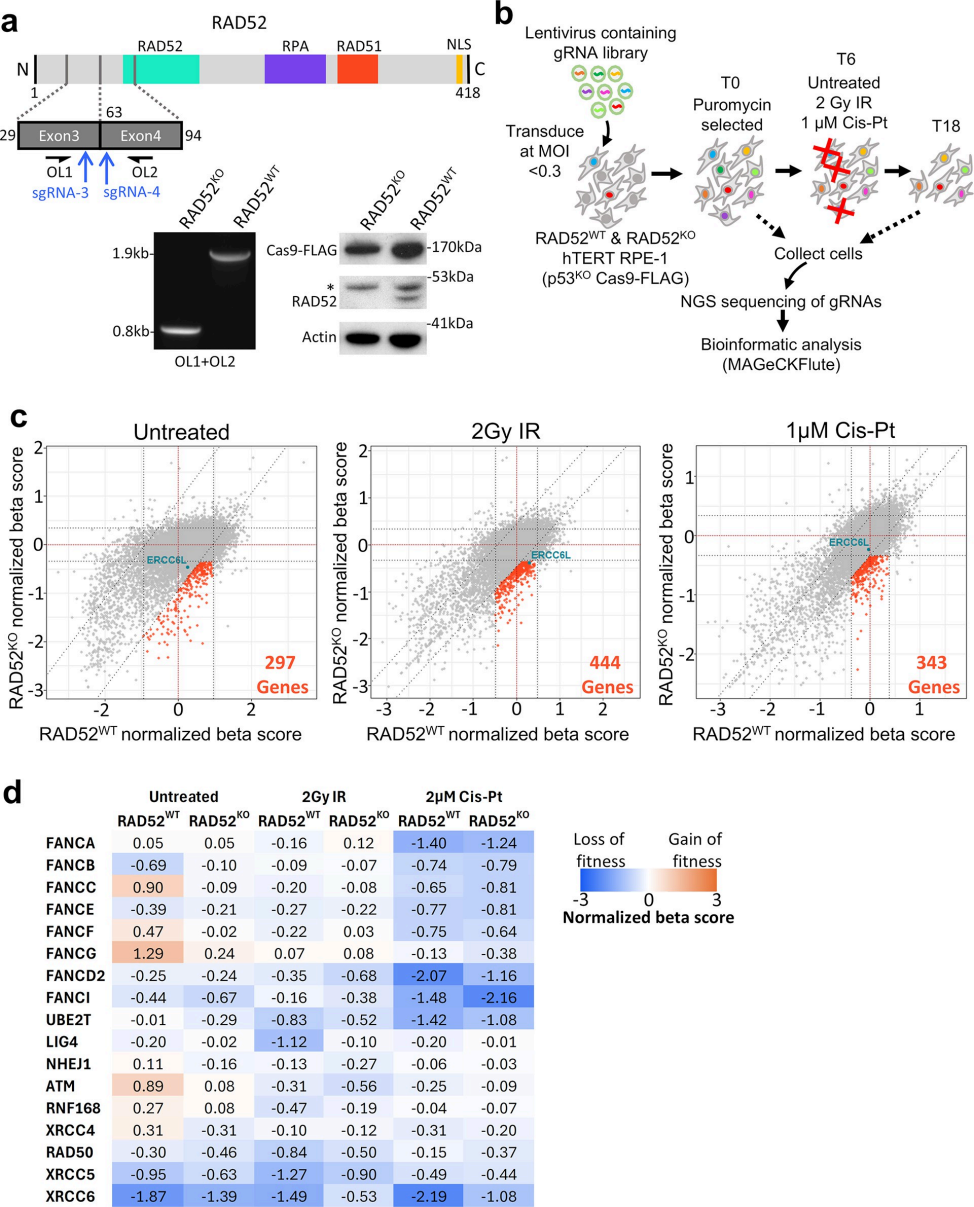
Genome-wide CRIPSR knockout screen identifies pathways that are synthetic lethal with RAD52. **a)** The RAD52 knockout (RAD52^KO^) cell line using p53^KO^ Cas9-FLAG-expressing RPE-1 hTERT (RAD52^WT^). Shown is a schematic of RAD52 with blue arrows depicting two sgRNAs used to generate a deletion in the *RAD52* gene, which was assessed by PCR using primers OL1 and OL2 (left gel), along with Immunoblot (right blot). *non-specific band. **b)** Schematic of genome-wide CRISPR knockout screen to identify RAD52 synthetic lethal interactors. **c)** Shown are synthetic lethal hits (orange data points) identified under each screening condition by applying cutoffs of 1.5x the standard deviation from the diagonal axis, x = 0 and y = 0 of the Beta (selectivity) scores generated by MAGeCKFlute. The IR screen hit, ERCC6L, is highlighted in teal to show its position on all three plots as it is the topic of downstream analysis later in this study. **d)** Heatmap of normalized beta scores for genes known to be linked to IR (NHEJ/ATM genes) and Cis-Pt (FA-pathway genes) sensitivity.

To identify genes and pathways that are synthetic lethal with RAD52, we performed genome-wide CRISPR-Cas9 knockout screens to measure loss of fitness for genes knocked out concurrently with RAD52. Namely, we sought to identify genes that when knocked out (KO) cause reduced fitness specifically in RAD52^KO^ cells as compared to RAD52^WT^ cells, which we refer to as the synthetic lethal hits. To this end, we generated a RAD52^KO^ cell line from a cell line used previously for DNA damage response screens (RPE-1 hTERT p53^KO^ Cas9) [44–46], referred to here as the parental or RAD52^WT^ cell line. Notably, these cell lines are p53^KO^ to facilitate survival of cells that undergo CRISPR-Cas9 gene editing [47], and because loss of p53 is relevant to many cancers [48]. We then confirmed knock-out of RAD52 in the RAD52^KO^ line by Immunoblot analysis (Fig 1A, see Materials & Methods for detailed description of cell line generation). Next, we performed a set of genome-wide CRISPR knockout screens to compare gene knockouts that affect the fitness of this RAD52^KO^ line as compared to its parental RAD52^WT^ line. During these screens, the cells were split into 3 treatment groups: 1) cells left untreated for 18 days after T0 (T18), 2) cells exposed to 2 Gy ionizing radiation (IR) on day T6, and then cultured for 12 days (total T18), and 3) cells treated with 1 μM Cisplatin (Cis-Pt) at T6 and for 6 days (T12), and then cultured for 6 more days (total T18) (Fig 1B). We performed three different treatment conditions to broaden the scope of our overall screen.

Next, we sequenced samples taken from the beginning (T0) and end (T18) timepoints for each cell line and condition, aligned sequencing reads to our reference list of library guides, and performed basic quality control analysis to ensure that sequencing produced a high guide mapping ratio (>85% for all samples) (S1 Fig). We then analyzed the resulting guide counts using the MAGeCK MLE algorithm [49] (Fig 1B and S2 Table, see Materials & Methods for full analysis details). This analysis produces a single selectivity score per gene (known as a beta score), which we then normalized to account for cell division rate differences between the RAD52^WT^ and RAD52^KO^ cell lines using the MAGeCKFlute analysis suite [50] (S2 Table). Each normalized beta score represents a loss of fitness (negative beta), gain of fitness (positive beta), or neutral (beta approaching zero) effect of a particular gene knockout.

Next, we plotted the normalized beta scores for all genes from the RAD52^WT^ screens (x-axis) against the RAD52^KO^ screens (y-axis) (Fig 1C). We applied cutoffs of 1.5-fold from the standard deviation to the x, y, and diagonal axes. From these cutoffs, we determined the “synthetic lethal hits” to be genes that are negatively selected in the RAD52^KO^ line, but non-selected in the RAD52^WT^ line (Fig 1C, orange sections). These “synthetic lethal hits” amounted to 297, 444, and 343 genes for the untreated, IR-treated, and Cis-Pt-treated screens respectively (Fig 1C and S2 Table). Combined, these screens produced 970 unique genes (i.e., genes not repeated between screens).

To initially assess the accuracy of the screens with respect to their differing treatments (Untreated, IR, and Cis-Pt), we inspected the normalized beta scores of factors known to be involved in the resolution of Cisplatin-induced interstrand crosslinks (Fanconi Anemia pathway) and those known to be involved in the DNA damage response to ionizing radiation (ATM and NHEJ). We hypothesized that the IR and Cis-Pt exposures inflicted during the screens would lead to a loss of fitness (as indicated by a negative normalized beta score) in cells from either cell line that received the guides for factors related to these pathways, as compared to cells that received the same guides in the untreated screen. We observed a strong loss-of-fitness effect from Cis-Pt treatment across all major Fanconi Anemia pathway genes queried (FANCA, FANCB, FANCC, FANCE, FANCF, FANCG, FANCD2, FANCI, and UBE2T) in both the RAD52^WT^ and RAD52^KO^ lines as compared to the Untreated screen (Fig 1D). In the RAD52^WT^ line, the effect of IR on fitness was similarly negative for cells with guides for LIG4, NHEJ1, ATM, RNF168, and XRCC4, although the effects were less pronounced in the RAD52^KO^ line (Fig 1D). Guides for RAD50, XRCC5, and XRCC6 negatively impacted fitness in both the untreated screen and the IR treated screen (Fig 1D). In total, these results indicate that the IR and Cis-Pt treatments, and subsequent screen analysis, identified genes known to be important for resistance to these treatments.

**Fig 2:**
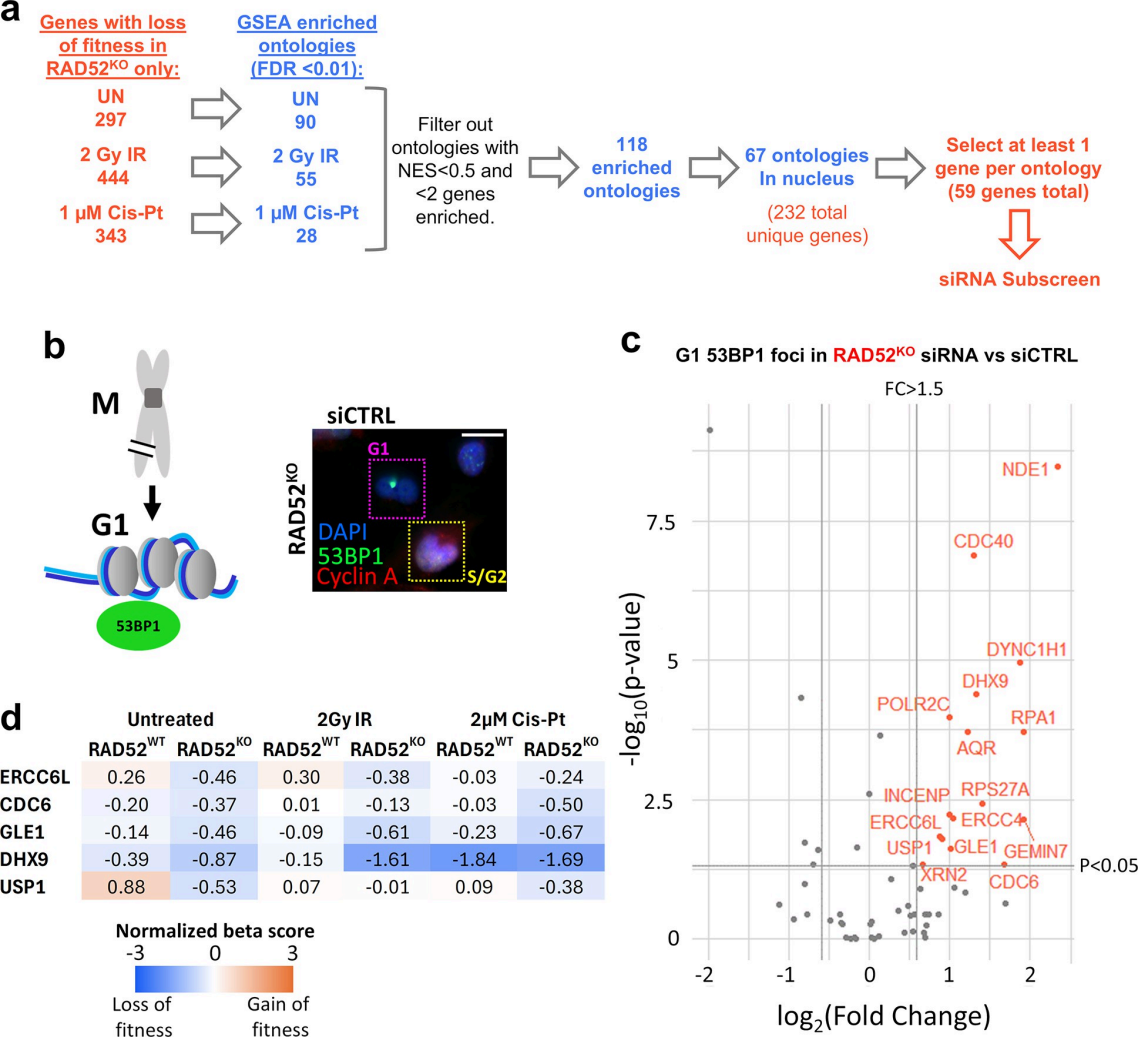
Secondary screen of 59 genes identifies 16 genes for which depletion causes G1-phase 53BP1 foci in RAD52^KO^ cells. **a)** Strategy for selecting 59 genes for secondary screening. Gene set enrichment analysis (GSEA) was performed on gene hits (orange text) from each screen independently and enriched ontologies (blue text) were selected based on false discovery rate (FDR) cutoff. Combined enriched ontologies from the 3 screens were then filtered by cutoffs for normalized enrichment score (NES) and minimum number of enriched genes. The 59 genes selected for the subscreen were chosen from the 67 ontologies (232 genes) that represent processes/pathways in the nucleus (at least 1 gene/ontology). **b)** 53BP1 foci assay. Shown is an illustration that DSBs that persist through mitosis (M) form G1 53BP1 nuclear bodies (left), along with a representative immunofluorescence image of 53BP1 and Cyclin A. Scale bar is 20 μm. Image taken at 20x magnification. **c)** Effects of siRNAs targeting the 59 genes (pool of 4 siRNAs per gene) in (a) on G1 53BP1 foci in RAD52^KO^ cells, compared to non-targeting control siRNA (siCTRL). Highlighted in red are the siRNAs that caused a significant, i.e., p<0.05 by Kolmogorov-Smirnov (K-S) test and >1.5-fold mean, increase in G1 53BP1 foci. N>50 total nuclei (G1 and S/G2) per siRNA. **d)** Heatmap of normalized beta scores from the genome-wide screens (Fig 1B) for the 5 hits selected by the siRNA subscreen.

Next, to identify a set of genes for secondary screening, we performed gene set enrichment analysis (GSEA) on the gene hits from each of the three screens separately (Fig 2A). The goal of this analysis approach was to organize gene hits into cellular pathways and processes (ontologies) important for cellular fitness with RAD52 loss. We queried multiple functional and pathway databases (e.g., Gene Ontology, Reactome, Kyoto Encyclopedia of Genes and Genomes) using the GSEA package included with MAGeCKFlute and imposed a cut off such that the false discovery rate (FDR) for each ontology was set at < 0.01. In doing so, we identified 90 (untreated), 55 (IR), and 28 (Cis-Pt) enriched ontologies (Fig 2A and S3 Table). We then filtered out any ontologies with fewer than 2 enriched genes or a normalized enrichment score (NES) of 0.5 or less, where a high NES indicates that enriched gene hits are distributed within known functional roles (as opposed to randomly) in an ontology (Fig 2A). This filtering led to 118 ontologies total (Fig 2A and S3 Table).

From these 118 filtered ontologies, we focused on the 67 that described pathways and processes relevant to the nucleus, because of the established role of RAD52 in DNA Repair [51] (Fig 2A and S3 Table). These nuclear ontologies contained a combined 232 unique gene hits, and we therefore sought to further narrow this set to select genes for secondary screening. We selected 59 gene hits to cover all 67 ontologies, with at least one gene selected from each ontology, and some genes present in multiple ontologies (Fig 2A and S3 Table, see Materials & Methods for further details on selection criteria).

### Secondary screen of 59 genes identifies 5, including ERCC6L/PICH, for which depletion causes G1-phase 53BP1 foci and reduced clonogenic survival in RAD52^KO^ cells

Given the role of RAD52 in DNA repair, we hypothesized that some of the screen hits may be involved in mitigating genotoxic stress, particularly when RAD52 is absent. One method to assay for genotoxic stress is to measure 53BP1 accumulation into foci, which occurs as part of the DNA damage response (DDR) [52]. More specifically, 53BP1 foci that form during G1-phase of the cell cycle (also known as G1 53BP1 nuclear bodies) appear to mark chromosomal breaks that originated from replication stress in the prior cell cycle that persisted through mitosis [53–55] (Fig 2B). Furthermore, a fraction of such G1 53BP1 foci persist into the subsequent S-phase where they are dissolved, presumably as damage is resolved to prevent further instability [56]. Thus, we sought to use G1 53BP1 foci (including both smaller foci and larger nuclear bodies) as an indicator of genotoxic stress that persists through mitosis. Meanwhile, we also sought to assess 53BP1 foci later in the cell cycle (S/G2-phase), which might indicate passage of such genotoxic lesions into S-phase, or response to new S-phase lesions.

Thus, to begin our secondary screening, we depleted each of the 59 of the selected genes described above (Fig 2A) in RAD52^KO^ and RAD52^WT^ cells by siRNA (pools of 4 siRNAs per gene) and examined 53BP1 foci by immunofluorescence microscopy. We chose to use siRNA depletion for the subscreen because isolation of double knockout clones with the RAD52^KO^ line may not be feasible for genes predicted to cause loss of fitness. Similarly, the process of isolating such cell lines may select for clones with adaptation responses that could mask important phenotypes. Thus, we used transient disruption of target genes, either with siRNA, or for some experiments with ERCC6L, transfection of Cas9/sgRNA complexes followed by analysis without long-term culturing or isolation of single clones. We also co-stained cells for Cyclin A to differentiate between S/G2-phase cells (Cyclin A positive) and G1-phase (Cyclin A negative) [54,55] (Fig 2B). Additionally, we included control cells that were treated with non-targeting siRNA (siCTRL). From the 59 genes analyzed (S4 Table), depletion of 16 produced significantly increased levels of G1 53BP1 foci (i.e., p<0.05, and a greater than 1.5-fold increase of the mean number of foci vs. siCTRL), in either RAD52^KO^ cells alone, or both RAD52^KO^ and RAD52^WT^ cells (Fig 2C, panels (a-b) in S2 Fig). Additionally, 17 of the 59 genes produced significantly increased levels of S/G2 53BP1 levels when depleted in the RAD52^KO^ or both lines (panels (c-d) in S2 Fig). Among these genes, 8 showed an increase in 53BP1 foci in both G1 and S/G2 (panels (b,d) in S2 Fig).

For the next phase of secondary screening to examine effects on clonogenic survival, we chose to focus on the 16 genes for which depletion caused an increase in G1 53BP1 foci for two reasons: 1) such foci appear to be an indicator of damage transmitted through mitosis [54,55,57] (although spontaneous lesions generated in G1 are also possible), and 2) RAD52 has been shown to have a role in DNA repair in mitosis, particularly for promoting mitotic DNA synthesis (MiDAS) [13–18]. Thus, we assessed effects of siRNAs targeting these 16 genes on viability in RAD52^KO^ cells and RAD52^WT^ by a clonogenic survival assay (normalized to parallel siCTRL treatment in each cell line) (S5 Table). We found that siRNAs targeting 5 genes (ERCC6L, CDC6, GLE1, DHX9, and USP1) caused a loss of viability that was significantly more severe in the RAD52^KO^ line as compared to the RAD52^WT^ line (panel (a) in S3 Fig). Moreover, when we inspected the normalized beta scores for these genes produced by the analysis from the initial genome-wide screens, we observed that all 5 genes exhibited a loss of fitness in the RAD52^KO^ line (more negative beta score) as compared to the RAD52^WT^ line under at least 2 of the 3 screening conditions (Fig 2D). Based on these findings, we confirmed siRNA depletion of ERCC6L, CDC6, GLE1, DHX9, and USP1 by Immunoblot (panel (b) in S3 Fig). In contrast, 7 of the remaining 11 genes (out of 16 total) tested by siRNA showed severe viability defects in both cell lines, making further study of a synthetic lethal interaction with RAD52 infeasible. Of the remaining 4 genes, 3 showed no significant difference between the two lines, and 1 gene improved viability in the RAD52^KO^ line compared to the RAD52^WT^ line (panel (a) in S3 Fig).

We also note that BLM, a factor previously described to act with RAD52 in processes such as MiDAS at telomeres [15], was identified in our initial screens as a hit, and indeed produced negative beta scores in the RAD52^KO^ line under all three screening conditions (panel (c) in S3 Fig). We performed analysis of siBLM in the subscreen, which included validation of the siRNA by immunoblotting (panel (d) in S3 Fig). Depletion of BLM did not have effects that met our screening criteria, and thus we did not proceed with studying the genetic interaction between BLM and RAD52.

### ERCC6L/PICH depletion causes elevated G1-phase 53BP1 foci and micronucleus formation, and reduced clonogenic survival in RAD52^KO^ cells

Among the genes identified above is ERCC6L (also known as PICH), which is a SWI/SNF2-family ATPase that interacts with the mitotic kinase PLK1, and has been shown to be critical for the resolution of DNA ultrafine bridges (UFBs) that form during anaphase of mitosis [32,58,59]. Because RAD52 is also implicated in genome stability in mitosis, for the remainder of this study we sought to further characterize the interrelationship between RAD52 and ERCC6L. To begin, we reviewed the results with ERCC6L from the initial genome-wide synthetic lethality screens. Indeed, normalized beta scores for ERCC6L were consistently negative across all three screening conditions in the RAD52^KO^ cell line (ranging from -0.24 to -0.46), while beta scores in the RAD52^WT^ line approached or exceeded 0 (ranging from -0.03 to +0.30) (Fig 2D). These findings indicate a consistent loss of fitness in each screen condition for ERCC6L in the RAD52^KO^ cell line vs. RAD52^WT^.

**Fig 3:**
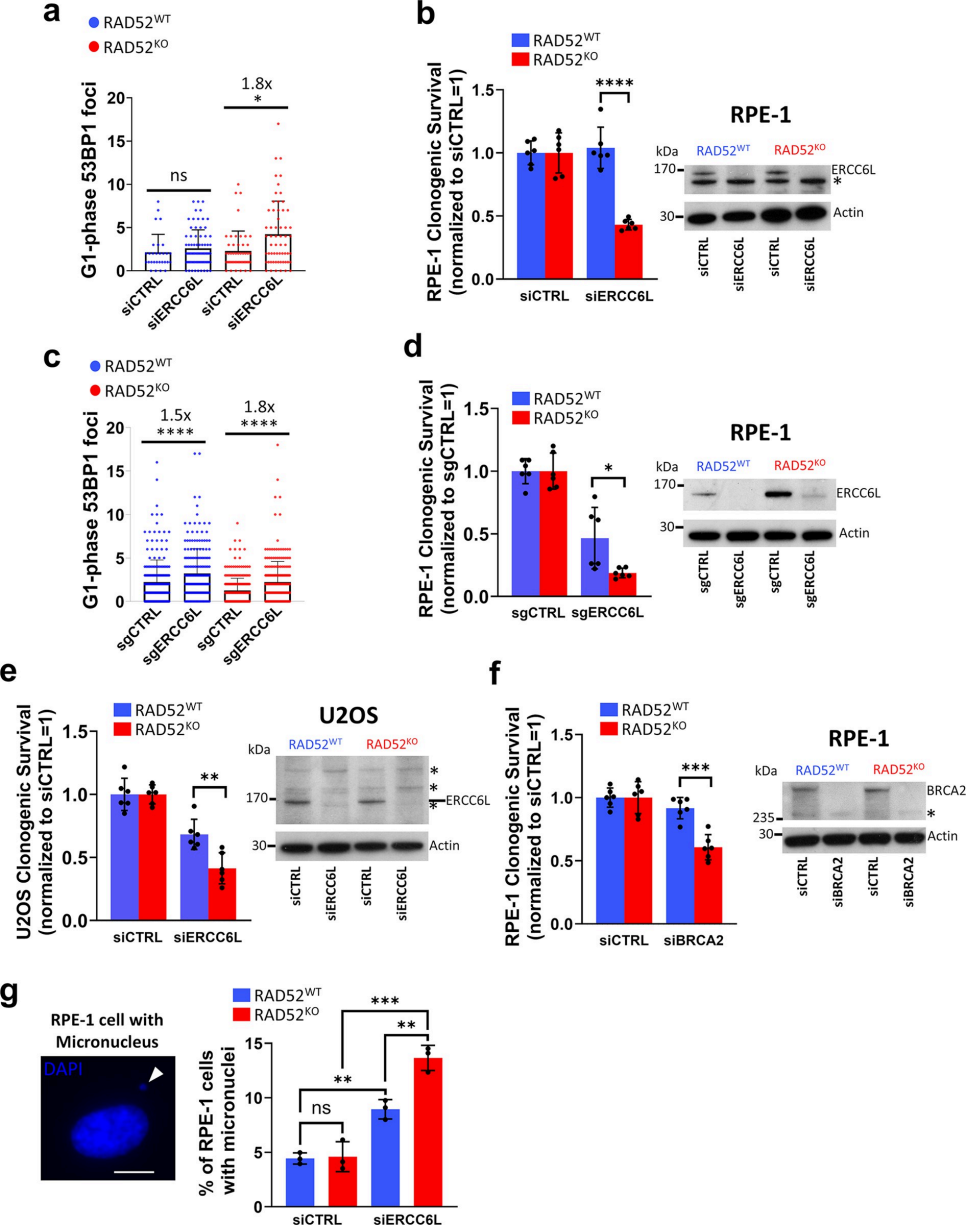
ERCC6L/PICH depletion causes elevated G1-phase 53BP1 foci and micronuclei formation, and reduced clonogenic survival in RAD52^KO^ cells. **a)** G1 53BP1 foci increase with depletion of ERCC6L by siERCC6L (pool of 4 siRNAs) treatment in RAD52^KO^, but not RAD52^WT^ RPE-1 cells. ns = not significant, * = p<0.05 by K-S test. Bars show mean foci value. Mean fold-increase is shown for significant comparisons. The total number of nuclei analyzed per condition was >50, with the number of G1 nuclei (N) analyzed per condition at N = 29–75. **b)** Clonogenic survival is reduced in RAD52^KO^ RPE-1 cells with depletion of ERCC6L by siERCC6L. Clonogenic survival after siERCC6L treatment is normalized to each respective siCTRL treated line (siCTRL = 1). **** = p<0.0001 by unpaired *t*-test. N = 6 replicates. Immunoblot confirming ERCC6L depletion via siERCC6L in RAD52^KO^ and RAD52^WT^ RPE-1 cell lines is shown at right. *non-specific band. **c)** G1 53BP1 foci increase with knock-down of ERCC6L by sgERCC6L treatment in RAD52^KO^ and RAD52^WT^ RPE-1 cells. **** = p<0.0001 by K-S test, bars show mean foci value. Mean fold-increase is shown for significant comparisons. Number of G1 nuclei (N) analyzed per condition is N = 214–276. **d)** Clonogenic survival and Immunoblot as in (b) but with ERCC6L knock-down by sgERCC6L. * = p<0.05 by unpaired *t*-test. N = 6 replicates. **e)** Clonogenic survival and Immunoblot as in (b) but in RAD52^KO^ and RAD52^WT^ U2OS cells. ** = p<0.01 by unpaired *t*-test. N = 6 replicates. **f)** Clonogenic survival and Immunoblot as in (b) but with BRCA2 depletion by siBRCA2 in RPE-1 cells. *** = p<0.001 by unpaired *t*-test. N = 6 replicates. **g)** (Left) Example of micronucleus in RPE-1 cell (white arrowhead). Scale bar is 10μm, and images were taken at 40x magnification (Right) Frequency of micronuclei increases with siERCC6L treatment in RAD52^KO^ cells, but not with siCTRL treatment in RAD52^KO^ cells as compared to RAD52^WT^. ns = not significant, ** = p<0.01, and *** = p<0.001 by unpaired *t*-test. N = 3 coverslips analyzed per condition, with at least 10 fields per coverslip.

We also focused on ERCC6L, because siRNA targeting of ERRC6L caused both an increase in G1 53BP1 foci, as well as a decrease in viability of RAD52^KO^ cells. To facilitate comparison with the below experiments, we first show this analysis with siERCC6L without also showing other genes (Fig 3A and 3B). As mentioned above, we found that siRNA targeting ERCC6L (siERCC6L) caused a significant (1.8-fold) increase in the mean number of G1 53BP1 foci compared to siCTRL in RAD52^KO^ cells, while there was no significant difference between ERCC6L depletion vs. siCTRL in RAD52^WT^ cells (Fig 3A). Moreover, siERCC6L treatment produced a significantly greater loss of clonogenic survival in RAD52^KO^ cells vs. RAD52^WT^ cells (Fig 3B). We also confirmed siRNA depletion of the ERCC6L protein by Immunoblot analysis in both the RAD52^KO^ and RAD52^WT^ cell lines (Fig 3B).

We then used other approaches to examine effects of ERCC6L and RAD52 on G1 53BP1 foci and clonogenic survival. First, we repeated the 53BP1 foci assay with CRISPR/Cas9-mediated knock-down of ERCC6L with sgRNAs targeting ERCC6L (sgERCC6L) in the RAD52^KO^ and RAD52^WT^ cell lines. Again, we saw a significant (1.8-fold) increase in mean G1 53BP1 foci after sgERCC6L treatment in RAD52^KO^ cells, while sgERCC6L treatment in RAD52^WT^ cells also produced a significant, but less severe (1.5-fold) increase as compared to sgCTRL treated cells (Fig 3C). Furthermore, treatment with sgERCC6L also produced a greater loss of clonogenic survival in RAD52^KO^ cells vs. RAD52^WT^ cells and we confirmed sgRNA-induced ERCC6L depletion by Immunoblot (Fig 3D). Next, we depleted ERCC6L in U2OS cell lines via treatment with siERCC6L and saw a similar loss of clonogenic survival in RAD52^KO^ U2OS cells as compared to RAD52^WT^ U2OS cells (Fig 3E). We also confirmed ERCC6L depletion in the U2OS cell lines by Immunoblot (Fig 3E).

In the experiments described above that were performed in RPE-1 cells, we noted that ERCC6L appeared to be expressed at a higher level in the RAD52^KO^ line as compared to the RAD52^WT^ line in the Immunoblot shown in Fig 2D. To investigate this further, we performed Immunoblots on replicates of the RAD52^WT^ and RAD52^KO^ RPE-1 cell lines, and quantified ERCC6L expression relative to actin (loading control) (panel (a) in S4 Fig). We did not observe a significant difference in ERCC6L expression among these replicates (panel (a) in S4 Fig).

Next, we performed a comparative analysis using BRCA2 depletion. While BRCA2 was not identified as a hit by our initial genome-wide screen (panel (c) in S4 Fig, see Discussion), it was previously reported that inactivation of RAD52 by shRNA is synthetic lethal in BRCA2-deficient cells [29]. Specifically, we treated our RPE-1 RAD52^WT^ and RAD52^KO^ cell lines with siRNA targeting BRCA2 (siBRCA2) and observed a comparable relative reduction in clonogenic survival for RAD52^KO^ vs. RAD52^WT^ (Fig 3F) to what we observed after siERCC6L treatment in these cell lines (Fig 3B). Notably, siBRCA2 also did not greatly impact viability in RAD52^WT^ cells, in contrast to studies showing BRCA2 is an essential gene [60,61]. To verify the siRNA reagent used in these experiments, we confirmed that siBRCA2 produced a homology directed repair (HDR) defect, using the DR-GFP reporter assay (previously described in [62]) (panel (b) in S4 Fig). We speculate that short-term depletion of BRCA2 via siRNA is not sufficient to cause the loss of viability that is observed with a genetic knockout.

Finally, we sought to assess micronuclei formation as another indicator of mitotic defects. We treated RAD52^WT^ and RAD52^KO^ RPE-1 cells with siERCC6L and observed significant increases in the frequency of cells with micronuclei in both RAD52^KO^ and RAD52^WT^ cells (Fig 3G). Importantly, siERCC6L-treated RAD52^KO^ cells had significantly higher levels of micronuclei as compared to siERCC6L-treated RAD52^WT^ cells (Fig 3G). By contrast, we observed no increase in micronuclei levels in RAD52^KO^ vs. RAD52^WT^ cells (siCTRL-treated, Fig 3G). Altogether, these findings indicate that depletion of ERCC6L in cells lacking RAD52 increases DNA damage and micronuclei formation, and negatively impacts fitness.

### RAD52 suppresses the accumulation of ERCC6L Ultra-Fine DNA bridges in anaphase

**Fig 4:**
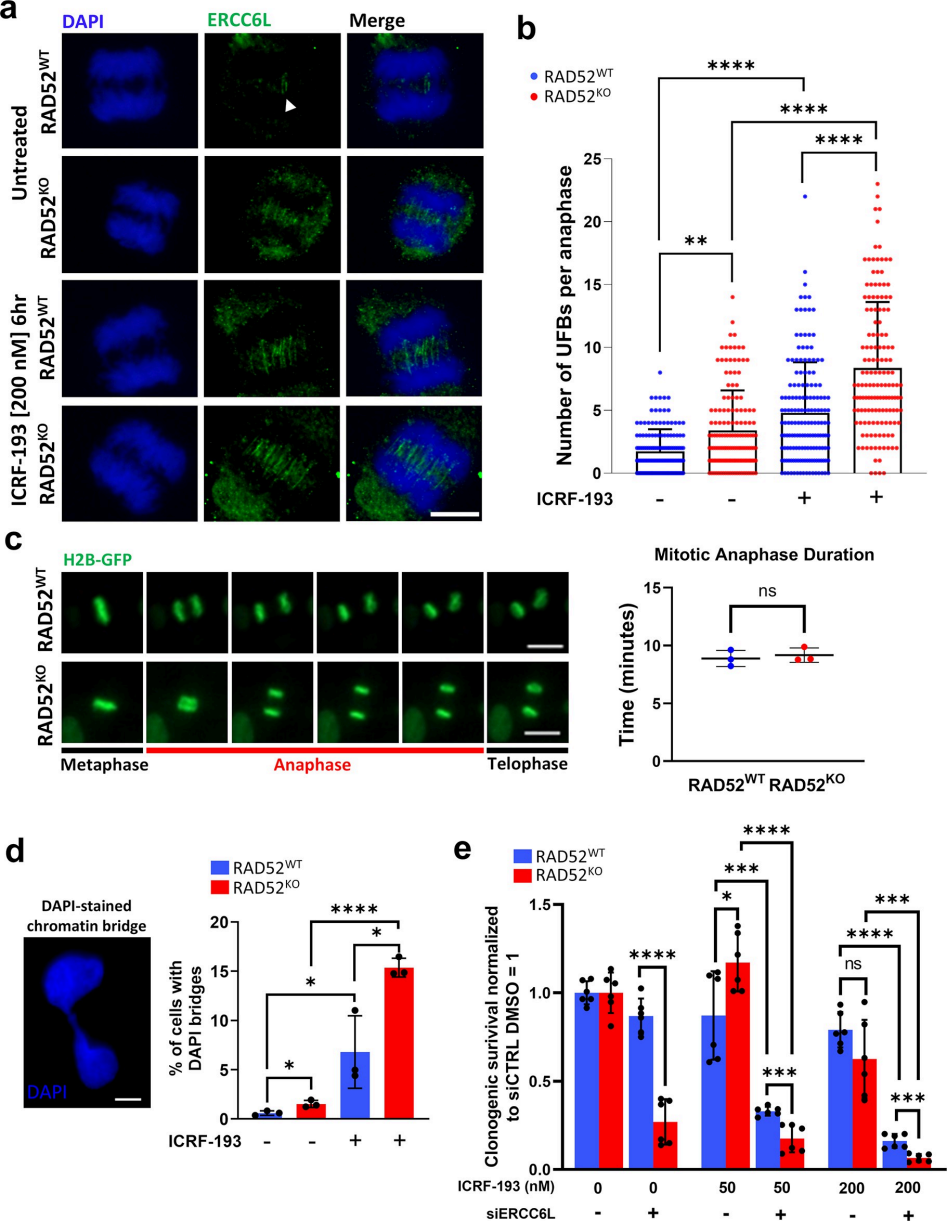
RAD52 suppresses the accumulation of ERCC6L Ultra-Fine DNA bridges in anaphase. **a)** ERCC6L UFBs in RPE-1 cells. Shown are examples of anaphase cells stained with ERCC6L for RAD52^WT^ and RAD52^KO^ cells with and without exposure to ICRF-193 [200 mM, 6-hour treatment]. White arrowhead indicates ERCC6L-UFBs in untreated RAD52^WT^ cells. Scale bar is 10 μm, and images were taken at 60x magnification. **b)** Anaphase ERCC6L-UFBs are increased in RAD52^KO^ cells as compared to RAD52^WT^ cells, which is amplified by exposure to ICRF-193 as shown in (a). Bars show mean value. The number of anaphases (N) analyzed per condition are N = 140–159. Significance determined by K-S test, ** = p<0.01, **** = p<0.0001. **c)** Live cell imaging of mitotic cells shows no significant difference in anaphase timing between RAD52^WT^ and RAD52^KO^ RPE-1 cells. (Left) Example of imaging of anaphase duration. Cells were labeled with H2B-GFP and imaged at a rate of 2min per frame. Scale bar is 20 μm, and images were taken at 20x magnification. (Right) Quantification of average anaphase duration for each cell line. Bars show average duration across N = 3 experiments, where at least 50 cells were analyzed per experiment. Significance determined by unpaired *t*-test. ns = not significant. **d)** (Left) Example of DAPI-staining chromatin bridge in RPE-1 cells. Images were taken at 20x magnification, and scale bar is 10μm. (Right) Frequency of DAPI/chromatin bridges increases in RAD52^KO^ cells as compared to RAD52^WT^ cells with DMSO and ICRF-193 treatment. * = p<0.05 and **** = p<0.0001 by unpaired *t*-test. N = 3 coverslips analyzed per condition, with at least 4 independent fields per coverslip. **e)** ICRF-193 exposure significantly impacts viability in siERCC6L-treated (pool of 4 siRNAs) RAD52^WT^ and RAD52^KO^ cells but does not significantly impact viability in RAD52^KO^ cells without siERCC6L. ICRF-193 treatments were for 55-hours. ns = not significant, * = p<0.05, *** = p<0.001, and **** = p<0.0001. Unpaired *t*-test. N = 6 replicates.

Based on the above findings, we then hypothesized that RAD52 and ERCC6L may have a compensatory relationship, i.e., that loss of one of these factors causes an increased reliance on the other. We sought to test this hypothesis using cell biology approaches. Namely, to begin to understand how ERCC6L might compensate for the loss of RAD52, we first tested whether the loss of RAD52 would cause an increase in ERCC6L-UFBs. As mentioned above, ERCC6L is known to play important roles in the resolution of anaphase UFBs at centromeres and late-replicating regions or as a result of replication stress [32,58,59], and ERCC6L itself localizes to such UFBs (i.e., ERCC6L-UFBs) [35,36]. Indeed, the identification of threads of ERCC6L in anaphase coincided with the discovery of UFBs, and as such, ERCC6L-UFBs are themselves a robust marker for UFBs [35,36]. Moreover, because ERCC6L staining is a hallmark of all UFBs, measurement of ERCC6L-UFBs likely reflects the frequency of total UFBs. Thus, we posited that without RAD52, cells may be more reliant on ERCC6L-UFBs for resolution of chromosomes in anaphase, as assessed by an increased frequency of these structures. To test this hypothesis, we performed immunofluorescence (IF) staining of the ERCC6L protein in RAD52^KO^ and RAD52^WT^ cells and quantified ERCC6L-UFBs in anaphase cells (Fig 4A and 4B). From this analysis, levels of ERCC6L-UFBs per anaphase were significantly increased in the RAD52^KO^ line as compared to the RAD52^WT^ line (Fig 4B).

Another factor important for mitigating UFBs is Topoisomerase IIα (Topo IIα), in that inhibition of Topo IIα by ICRF-193 increases the frequency of ERCC6L-UFBs [35,63,64]. Thus, we tested whether loss of RAD52 may also cause an increase in ERCC6L-UFBs caused by Topo IIα inhibition. For this, we treated cells with 200 nM ICRF-193 for 6 hours prior to fixation and IF staining, and we found that ICRF-193 treatment significantly increased ERCC6L-coated UFBs in both the RAD52^KO^ and RAD52^WT^ lines as compared to their untreated counterparts, however the most severe effect was observed in the RAD52^KO^ line (Fig 4A and 4B).

In these analyses we included all anaphases because we found anaphase staging (e.g., distinguishing early vs. late anaphase) difficult in RPE-1 cells, perhaps because anaphase is completed relatively quickly. Thus, we performed an additional control experiment to examine anaphase timing. Specifically, we sought to test whether anaphase timing was distinct between the RAD52^KO^ and RAD52^WT^ cell lines. For this analysis, we performed live cell imaging experiments to measure anaphase duration and found that there was no significant difference in anaphase duration between RAD52^KO^ and RAD52^WT^ cells (Fig 4C). We also measured timings of prometaphase and metaphase in these experiments and found that only metaphase duration was modestly decreased in the RAD52^KO^ line as compared to RAD52^WT^ (S5 Fig). These findings indicate that loss of RAD52 increases the frequency of anaphase ERCC6L-UFBs both under unstressed/spontaneous conditions, and with Topo IIα inhibition. Moreover, loss of RAD52 does not impact anaphase timing.

Next, we assessed whether in addition to increased UFB levels, we could also observe elevated chromatin bridges in interphase cells (i.e., after mitosis), which can be detected by DAPI staining. In Rad52^KO^ cells, we observed significant increases in the frequencies of interphase cells with DAPI-staining chromatin bridges compared to Rad52^WT^ cells (Fig 4D). Furthermore, when we treated cells with ICRF-193 for 6 hours prior to fixation, we also observed a significant increase in DAPI-staining bridges in the Rad52^KO^ line vs. Rad52^WT^ (Fig 4D). These results mirror our ERCC6L-UFB results between Rad52^KO^ and Rad52^WT^ cells. Thus, loss of RAD52 not only causes elevated ERCC6L-UFBs, but also causes DNA bridges that persist into interphase, altogether supporting a role for RAD52 in mitosis.

Finally, we tested the effects of combined depletion of ERCC6L and ICRF-193 treatment on clonogenic survival in RAD52^KO^ cells. We treated RAD52^KO^ and RAD52^WT^ cells with 50 nM or 200 nM ICRF-193 after transfection with siERCC6L or siCTRL (i.e., a 2-day ICRF-193 treatment, which was then removed, and followed by assessment of colony formation). For both concentrations of ICRF-193, RAD52^KO^ cells did not show a significant decrease in clonogenic survival vs. RAD52^WT^ cells (treated with siCTRL) (Fig 4E). In contrast, a combination treatment of siERCC6L with ICRF-193 caused a decrease in clonogenic survival with both RAD52^KO^ and RAD52^WT^ cells, but with a greater decrease in RAD52^KO^ cells (Fig 4E). This marked loss of viability was most severe in RAD52^KO^ cells treated with 200nM ICRF-193 and siERCC6L (Fig 4E). These findings indicate that RAD52 is important for survival following disruption of two factors important for resolving replication stress at mitosis (i.e., Topo IIα and ERCC6L).

### RAD52 forms foci in prometaphase cells in response to ERCC6L depletion

**Fig 5:**
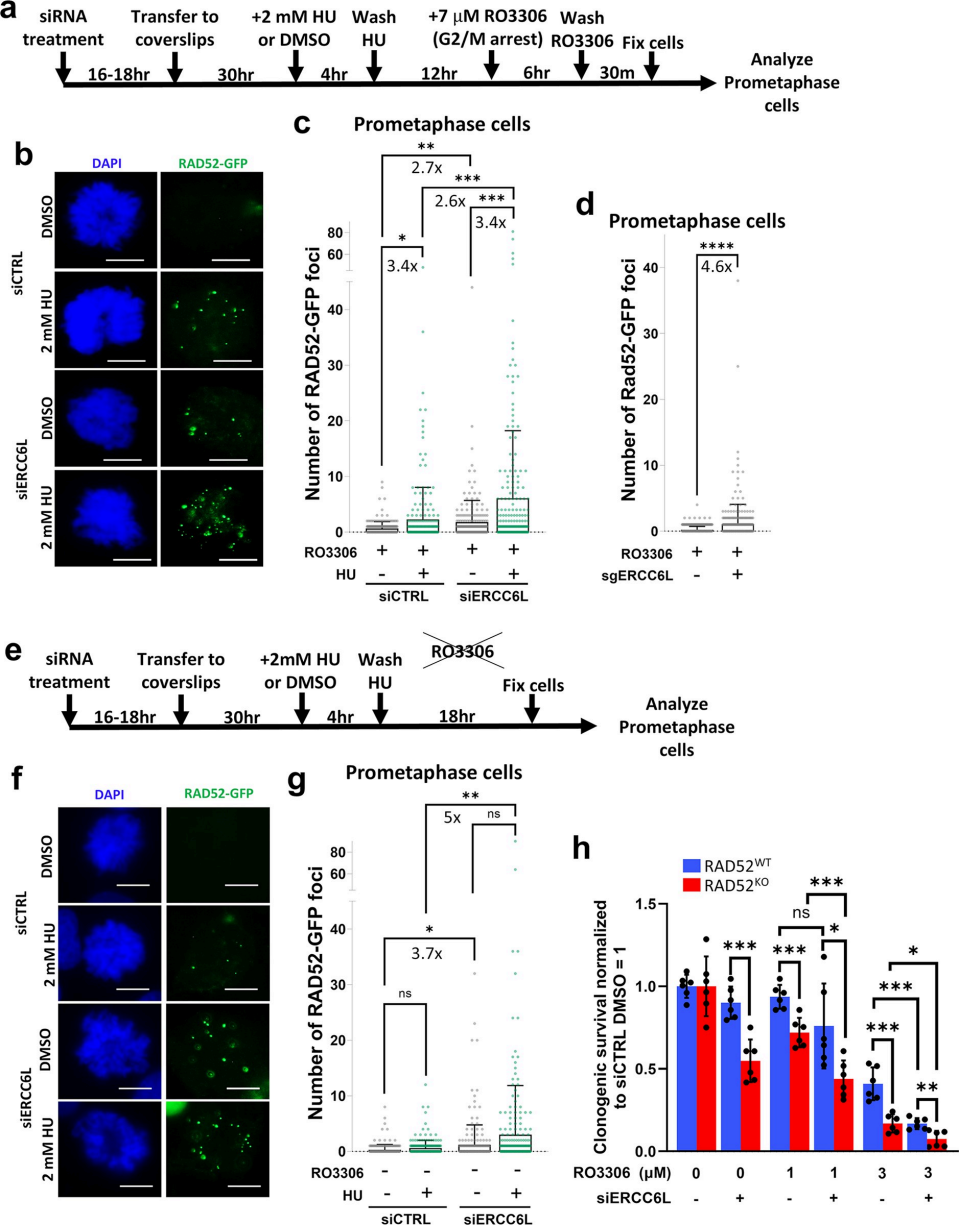
RAD52 forms foci in prometaphase cells in response to ERCC6L depletion. **a)** Schematic of treatments used to examine RAD52-GFP foci in prometaphase cells. **b)** Shown are representative images of prometaphase cells with RAD52-GFP foci, after treatments as shown in (a). Scale bars are 10 μm, and images were taken at 40x magnification. **c)** Treatments with siERCC6L (pool of 4 siRNAs) and HU each induce significant increases in RAD52-GFP foci in prometaphase cells. Cells were treated as in (a) and analyzed as in (b). Bars indicate mean foci values, and fold increases are shown. * = p<0.05, ** = p<0.01, and *** = p<0.001, K-S test. The number of nuclei (N) analyzed per condition are N = 210–218. **d)** Treatment with CRISPR-Cas9 guides targeting ERCC6L (sgERCC6L, pool of 3 guides) induces a similar increase in RAD52-GFP foci as seen in (c). Cells were treated as in (S6A Fig). Bars show mean foci values, and fold increase is indicated. **** = p<0.0001, K-S test. The number of nuclei (N) analyzed per condition are N = 323–336. **e)** Schematic of treatments to examine RAD52-GFP foci without using the CDK1 inhibitor RO3306 (i.e., no induction of G2/M arrest). **f)** Shown are representative images of RAD52-GFP foci from the treatments as shown in (e). Scale bars are 10 μm and images were taken at 40x magnification. **g)** Without RO3306-induced G2/M arrest, RAD52-GFP foci are increased with siERCC6L treatment, but not HU treatment. Cells were treated as in (e) and analyzed as in (f). Shown are mean foci values and fold increases. N = 225–237. ns = not significant, * = p<0.05, ** = p<0.01, K-S test. **h)** RO3306 exposure significantly impacts survival in RAD52^KO^ cells with and without ERCC6L depletion, and ERCC6L-depleted RAD52^WT^ cells at the high (3 μM), but not low (1 μM) concentration. Clonogenic survival assay was performed with two concentrations of RO3306 (1 μM or 3 μM) for 48 hours in RAD52^WT^ and RAD52^KO^ cell lines. Clonogenic survival is normalized to DMSO and siCTRL treated RAD52^WT^ and RAD52^KO^ cell lines (DMSO siCTRL = 1). ns = not significant, * = p<0.05, ** = p<0.01, *** = p<0.001, unpaired *t*-test. N = 6 replicates.

Given that loss of RAD52 appears to cause an increased reliance on ERCC6L-UFBs in anaphase, we next sought to test the converse hypothesis, i.e., that cells may rely more heavily on RAD52 when ERCC6L is depleted, particularly in mitosis. To test this, we stably transfected hTERT RPE-1 P53^KO^ cells with GFP-tagged RAD52 (RAD52-GFP) to examine RAD52 focal accumulation, as previously described [65]. We treated these cells with siERCC6L vs. siCTRL, and then exposed cells to replication stress via treatment with hydroxyurea (HU) that depletes nucleotide pools (4-hour treatment with 2 mM HU, followed by 12-hour recovery). We also performed a mock treatment without HU (DMSO control). Following this treatment, we enriched for mitotic cells by treating cells with the CDK1 inhibitor RO3306 at a concentration of 7 μM for 6 hours to synchronize cells in G2/M, and then released cells into mitosis. 30 minutes after the release, cells were fixed, and we assessed RAD52-GFP foci by IF microscopy in prometaphase cells (Fig 5A). We observed marked increases in RAD52-GFP foci in siERCC6L treated cells as compared to siCTRL treated cells both with and without HU exposure (Fig 5B and 5C). Additionally, cells treated with HU produced significantly increased RAD52-GFP foci levels as compared to their untreated counterparts (Fig 5C).

We then used another approach to confirm the effects of ERCC6L depletion on RAD52-GFP foci formation. We performed CRISPR/Cas9-mediated knock-down of ERCC6L with sgRNAs targeting ERCC6L (sgERCC6L) or with non-targeting sgRNAs (sgCTRL). Six days after transfection, cells were synchronized using RO3306, similar to the experiments shown in Fig 5A–5C (See S6 Fig for full schematic of experiment). From these experiments, we saw a significant increase in RAD52-GFP foci after CRISPR/Cas9-mediated ERCC6L knock-down, recapitulating our results from experiments using siRNA. Altogether, these results indicate that RAD52 localizes to prometaphase cells in response to ERCC6L depletion.

While RO3306 treatment is a common approach to enrich prometaphase cells, recent studies have indicated that such treatment can affect the cellular response to replication stress [66]. Thus, we wondered if RO3306 treatment may influence the effect of ERCC6L depletion and/or HU treatment on RAD52-GFP foci formation. Thus, we assessed RAD52-GFP foci in cells treated with siERCC6L, with and without HU treatment, but without the addition of RO3306 (Fig 5E). We quantified RAD52-GFP foci in prometaphase cells and found that siERCC6L treatment caused a significant increase in RAD52-GFP foci as compared to siCTRL (Fig 5F and 5G), similar to the experiments with RO3306 treatment (Fig 5B and 5C). In contrast, RAD52-GFP foci were not significantly increased with the addition of HU as compared to untreated cells, both for siCTRL and siERCC6L treated cells (Fig 5G). Thus, ERCC6L depletion causes RAD52-GFP foci in prometaphase cells irrespective of induction of G2/M arrest via RO3306, whereas HU only induces such RAD52-GFP foci when combined with RO3306 treatment.

Based on these findings, we then considered that RO3306 treatment may activate RAD52-dependent repair. Namely, we posited that RO3306-treated cells may be more reliant on RAD52, which we assessed with clonogenic survival after 48-hour treatment with 1 μM or 3 μM RO3306. In cells treated with either concentration of RO3306, we observed that clonogenic survival was indeed significantly decreased in RAD52^KO^ cells as compared to RAD52^WT^ cells (siCTRL-treated), with the 3 μM RO3306 treatment having the greatest impact (Fig 5H). Next, we assessed whether siERCC6L treatment would have a similar impact on clonogenic survival in RO3306-treated cells. Beginning with RAD52^WT^ cells, 3 μM RO3306 treatment (but not 1 μM RO3306 treatment), induced a significant decrease in clonogenic survival when combined with siERCC6L treatment (Fig 5H). With RAD52^KO^ cells, treatment with siERCC6L caused a further decrease in viability when combined with both 1 μM and 3 μM RO3306 exposure (Fig 5H). Altogether, these findings indicate that ERCC6L depletion induces RAD52-GFP foci in prometaphase irrespective of CDK1 inhibition (i.e., RO3306 treatment), and that cells also rely more heavily on RAD52 when CDK1 is inhibited (causing G2/M arrest).

### RAD52-GFP foci induced by ERCC6L depletion are distinct from sites of mitotic DNA synthesis (MiDAS)

**Fig 6:**
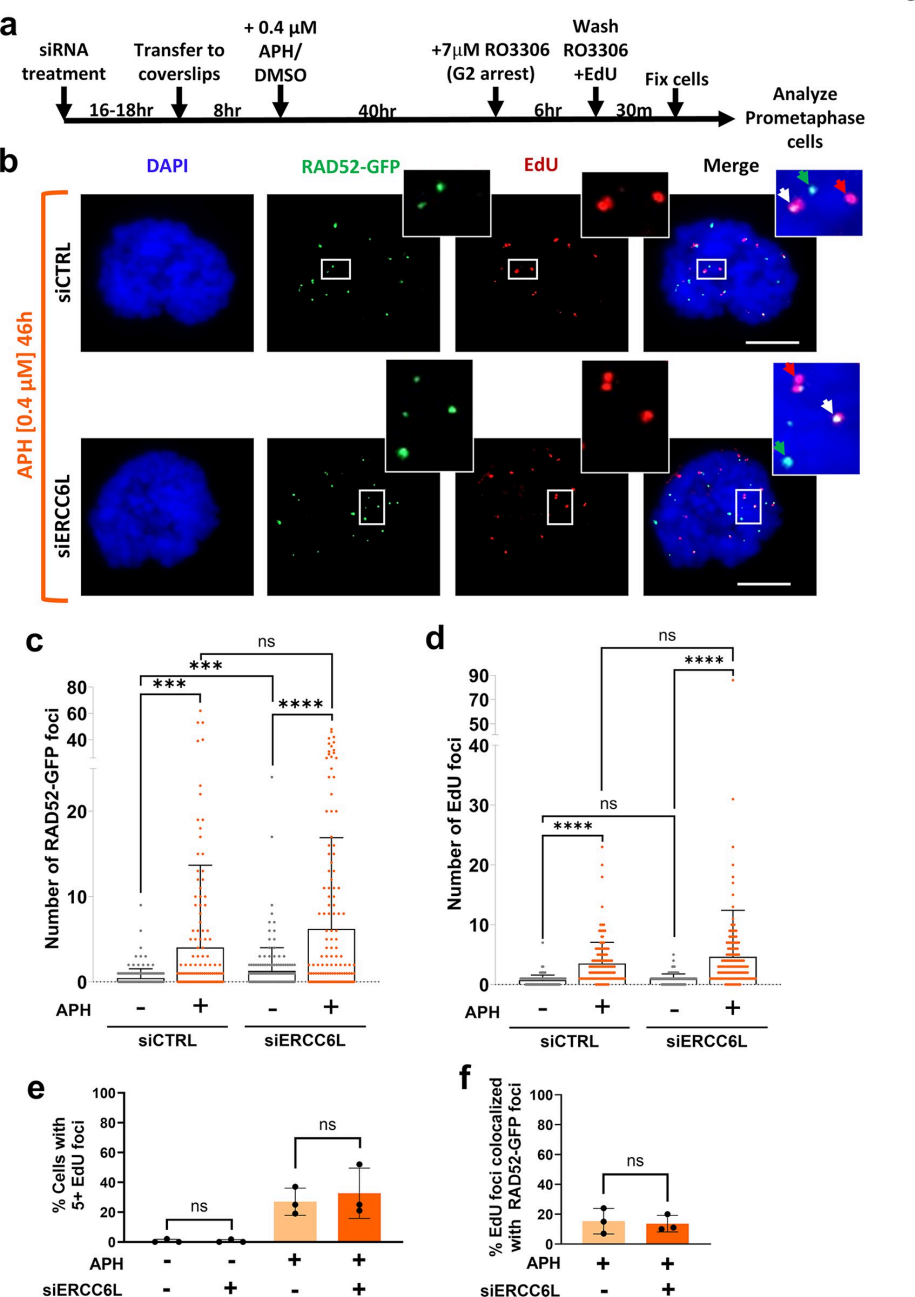
RAD52 foci induced by ERCC6L depletion are distinct from sites of mitotic DNA synthesis (MiDAS). **a)** Schematic of treatments used for MiDAS detection in prometaphase cells expressing RAD52-GFP. **b)** Shown are representative images of APH-treated cells with RAD52-GFP and EdU foci. Merge and zoom images show individual RAD52-GFP (green arrow), EdU (red arrow), and colocalized foci (white arrow). Scale bars are 10 μm, and images were taken at 40x magnification. **c)** RAD52-GFP foci are induced by treatments with aphidicolin (APH, 46 hours) and siERCC6L (pool of 4 siRNAs), but are not further increased with combined treatment of siERCC6L and APH. Treatments were performed as in (a). Bars show mean foci value. The number of nuclei (N) analyzed per condition are N = 158–162. ns = not significant, *** = p<0.001, and **** = p<0.0001, K-S test. **d)** EdU foci increase with APH treatment, but not with siERCC6L treatment. Treatments were as in (a). Bars show mean foci value. N = 158–162. ns = not significant, **** = p<0.0001, K-S test. **e)** No significant difference in the percentage of cells with 5 or more EdU foci between siCTRL and siERCC6L treated cells after APH exposure. Analysis of the data shown in (c) and (d). N = 3 independent experiments. ns = not significant, unpaired *t*-test. **f)** The percentage of EdU foci that colocalize with RAD52-GFP foci in cells that have ≥5 EdU foci is not significantly different in cells treated with siCTRL or siERCC6L after APH exposure. Analysis of the data shown in (c) and (d). N = 3 independent experiments. ns = not significant, unpaired *t*-test.

RAD52 has been proposed to promote Mitotic DNA Synthesis (MiDAS), which is typically induced by extended treatment of cells with the DNA polymerase α inhibitor Aphidicolin (APH) [58,67]. Thus, we sought to define the relationship between RAD52-GFP foci and sites of MiDAS under various conditions. In particular, we compared effects of siERCC6L vs. APH treatment on both RAD52-GFP foci and induction of MiDAS. The assay for MiDAS involves detecting incorporation of the nucleoside analogue 5-ethynyl-2′-deoxyuridine (EdU) during mitosis [68]. Furthermore, MiDAS is commonly assessed using release from RO3306 treatment [13,16,68], which we also included in this analysis. Specifically, we treated cells with siERCC6L or siCTRL (all cells without siERCC6L treatment were treated with siCTRL), then exposed cells to 0.4 μM APH for 46 hours, synchronized cells with RO3306 in the last 6 hours of APH exposure, released cells into media containing EdU for 30 minutes prior to fixation, and analyzed prometaphase cells by IF to detect EdU and RAD52-GFP (Fig 6A and 6B). From this analysis, we found that APH treatment induced significant increases in RAD52-GFP foci (Fig 6C). In addition, siERCC6L treatment also caused an increase in RAD52-GFP foci (Fig 6C), similar to results described above for prometaphase cells without APH treatment (Fig 5C and 5G). However, combining APH and siERCC6L treatment did not induce a significant increase vs. APH-treatment alone (Fig 6B and 6C). We next quantified EdU foci and found that APH treatment produced significantly higher levels of EdU foci compared to untreated cells (Fig 6D), but that siERCC6L treatment did not cause an increase in EdU foci, neither with nor without APH treatment (Fig 6B and 6D). We also examined cells with 5 or more EdU foci to specifically examine conditions that produced cells with relatively high levels of MiDAS and found that only cells with APH treatment produced such elevated EdU foci levels, with no effect from siERCC6L treatment (Fig 6E). For comparison, we also examined HU treatment conditions that caused RAD52-GFP foci (panel (a) in S7 Fig) and found that such treatment did not produce a significant increase in EdU foci as compared to untreated cells, regardless of siERCC6L treatment (panel (b) in S7 Fig). In summary, while both APH treatment and siERCC6L treatment cause RAD52-GFP foci in prometaphase cells, the combined treatment of APH and siERCC6L did not produce higher levels of RAD52-GFP foci vs. APH alone, and only APH treatment caused an increase in MiDAS.

In addition, we tested whether EdU foci colocalized with RAD52-GFP foci, and whether such colocalization was affected by siERCC6L treatment. Specifically, we used APH-treated cells with high levels of MiDAS and examined computed distances between foci maxima, where we considered two maxima to be colocalized if they were less than 3 pixels (~0.34 μm) apart. For cells treated with siCTRL, we found that RAD52-GFP foci and MiDAS (EdU foci) colocalize relatively infrequently (14–15% EdU with RAD52-GFP foci, 19–22% RAD52-GFP with EdU foci) (Fig 6B and 6F, panel (c) in S7 Fig). Furthermore, we found that there was no significant difference between siERCC6L-treated cells and siCTRL for such colocalization (Fig 6B and 6F, panel (c) in S7 Fig). Altogether, these data indicate that although RAD52 localizes in prometaphase cells in response to ERCC6L depletion, HU and APH exposure, this localization is not often associated with EdU incorporation (sites of DNA synthesis). Thus, it is unlikely that MiDAS is the main compensatory mechanism in which RAD52 functions to mitigate genotoxic stress induced by ERCC6L depletion or replication stress.

### Replication Stress induces delayed RAD52-GFP foci in interphase cells with ERCC6L depletion

**Fig 7:**
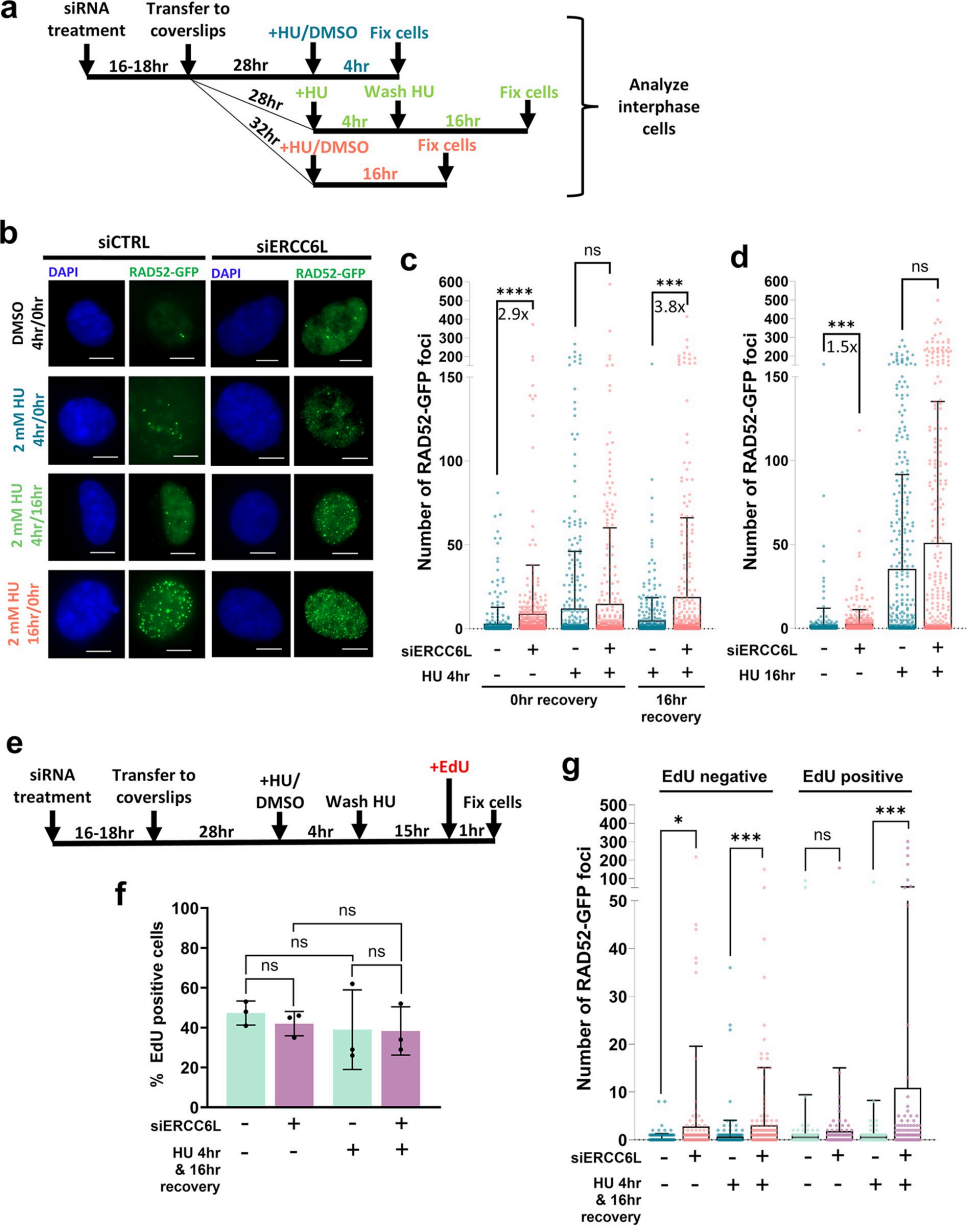
Replication Stress induces delayed RAD52-GFP foci in interphase cells with ERCC6L depletion. **a)** Schematic of treatments to examine RAD52-GFP foci in interphase cells. **b)** Shown are representative images of interphase cells to detect RAD52-GFP foci. Treatments were performed as shown in (a). Scale bars are 10 μm and images were taken at 40x magnification. **c)** RAD52-GFP foci in interphase cells significantly increase with siERCC6L treatment (pool of 4 siRNAs) both without HU treatment, and when combined with a 4 hr HU treatment followed by recovery for 16 hr. Bars show mean foci value, and fold-increases are shown. The number of nuclei (N) analyzed per condition are N = 372–462. ns = not significant, *** = p<0.001, and **** = p<0.0001, K-S test. **d)** RAD52-GFP foci in interphase cells are significantly increased by 16-hour HU exposure, which is not further increased with siERCC6L treatment. Bars and fold-increases are as in (c). N = 349–380. ns = not significant, *** = p<0.001, and **** = p<0.0001, K-S test. **e)** Schematic of experiment to examine RAD52-GFP foci in interphase cells with EdU labeling after DMSO or HU treatment and recovery. **f)** siERCC6L and HU treatments shown in (e) do not alter the percentage of cells undergoing DNA synthesis (EdU-positive cells). ns = not significant, unpaired *t*-test. N = 3 independent coverslips analyzed. **g)** RAD52-GFP foci in interphase cells significantly increase with siERCC6L treatment in EdU-negative cells with and without HU treatment but are only significantly increased in EdU-positive cells after HU treatment and recovery. Bars show mean foci value. The number of nuclei (N) analyzed per condition are N = 330–342. ns = not significant, * = p<0.05, and *** = p<0.001, K-S test.

To understand how RAD52 might further mitigate genotoxic stress outside of mitosis, we next asked if ERCC6L depletion might also alter RAD52 localization in interphase cells. Specifically, we hypothesized that in addition to RAD52 recruitment in mitosis in response to ERCC6L depletion, unresolved replication stress in the subsequent interphase associated with ERCC6L depletion may also recruit RAD52. To test this, we treated our RAD52-GFP cell line with siERCC6L and examined RAD52-GFP foci in interphase cells (Fig 7A, DMSO treatment). We observed a significant increase in RAD52-GFP foci after siERCC6L treatment at either of the two time points (i.e., fixation 48 hours (2.9-fold increase) and 64 hours (1.5-fold increase) after transfection, Fig 7B–7D, respectively, samples without HU). This result indicates that ERCC6L depletion causes RAD52-GFP foci in interphase. Given the key role that ERCC6L plays in mitosis, we suggest that interphase RAD52-GFP foci are caused by defects that are transmitted from the prior mitosis, although it is possible that ERCC6L is also playing a role in interphase to suppress these RAD52-GFP foci.

Next, we sought to examine how replication stress timing (i.e., treatment and recovery timing) might affect the emergence of interphase RAD52-GFP foci. Using a series of HU treatments that varied in length, and with or without recovery periods, we measured RAD52-GFP foci in response to siERCC6L or siCTRL treatment (Fig 7A and 7B). In cells treated with HU for 4 hours, but that were allowed to recover for 16 hours (16 hr recovery), we observed significantly increased (3.8-fold) levels of RAD52-GFP foci with siERCC6L treatment (Fig 7C). By contrast, cells that were treated with HU for 4 hours without recovery (0 hr recovery), or 16 hours without recovery, showed no significant difference in RAD52-GFP foci between siERCC6L and siCTRL treatments (Fig 7C and 7D, respectively). As a control, we examined effects of siERCC6L treatment on the proportion of cells in S-phase/G2, as compared to siCTRL. Specifically, we performed IF staining for Cyclin A expression to assess the percentage of cells in S/G2-phase for each treatment condition shown in Fig 7A. (panel (a) in S8 Fig). We observed that siERCC6L treatment did not substantially change the proportion of Cyclin A positive cells (S/G2-phase cells), either on its own, or under any HU treatment condition (panel (b) in S8 Fig). In summary, the combined effect of HU treatment and siERCC6L treatment on RAD52-GFP foci required a recovery phase after the HU treatment. We suggest that the requirement of a long recovery time after HU exposure to increase RAD52-GFP foci reflects the additive effect of genotoxic stress caused by replication stress and ERCC6L depletion in the prior cell cycle. Although, as above, we cannot exclude the possibility that such replication stress occurred in the same cell cycle phase as the foci formation.

Finally, we tested whether cells with high levels of RAD52-GFP foci due to siERCC6L treatment represented cells that were in S-phase and/or G2 of the cell cycle. Specifically, we assessed whether the RAD52-GFP foci in these experiments were associated with cells undergoing replication, which we marked with EdU labelling. We repeated the experiment shown in Fig 7A–7D for the 4-hour HU or DMSO treatment conditions with 16 hours of recovery, but prior to fixation, we first incubated cells in media containing EdU for 1 hour (Fig 7E). We saw similar effects of siERCC6L and HU treatment on RAD52-GFP foci levels as observed in prior experiments (panel (c) in S8 Fig), and the percentage of EdU-positive cells was similar among all samples, regardless of siERCC6L or HU treatment status (Fig 7F). Interestingly, we found that RAD52-GFP foci increased after siERCC6L treatment only with EdU-negative cells (Fig 7G). In contrast, combining siERCC6L and HU treatment/recovery caused elevated RAD52-GFP foci in both EdU-negative and EdU-positive cells (S-phase cells, Fig 7G, panel (d) in S8 Fig). The RAD52-GFP foci in EdU-negative cells likely represent localization to replication stress that has persisted into G2 phase, due to the high percentage of such cells that stain with Cyclin A (80%, panel (b) in S8 Fig). These findings indicate that the RAD52-GFP foci in interphase cells caused by ERCC6L depletion are not associated with S phase, whereas combining ERCC6L depletion and HU treatment/recovery causes RAD52 foci in both S and G2 phase cells.

### ERCC6L depletion induces RAD51 foci in interphase cells after HU exposure that colocalize with RAD52-GFP foci

**Fig 8:**
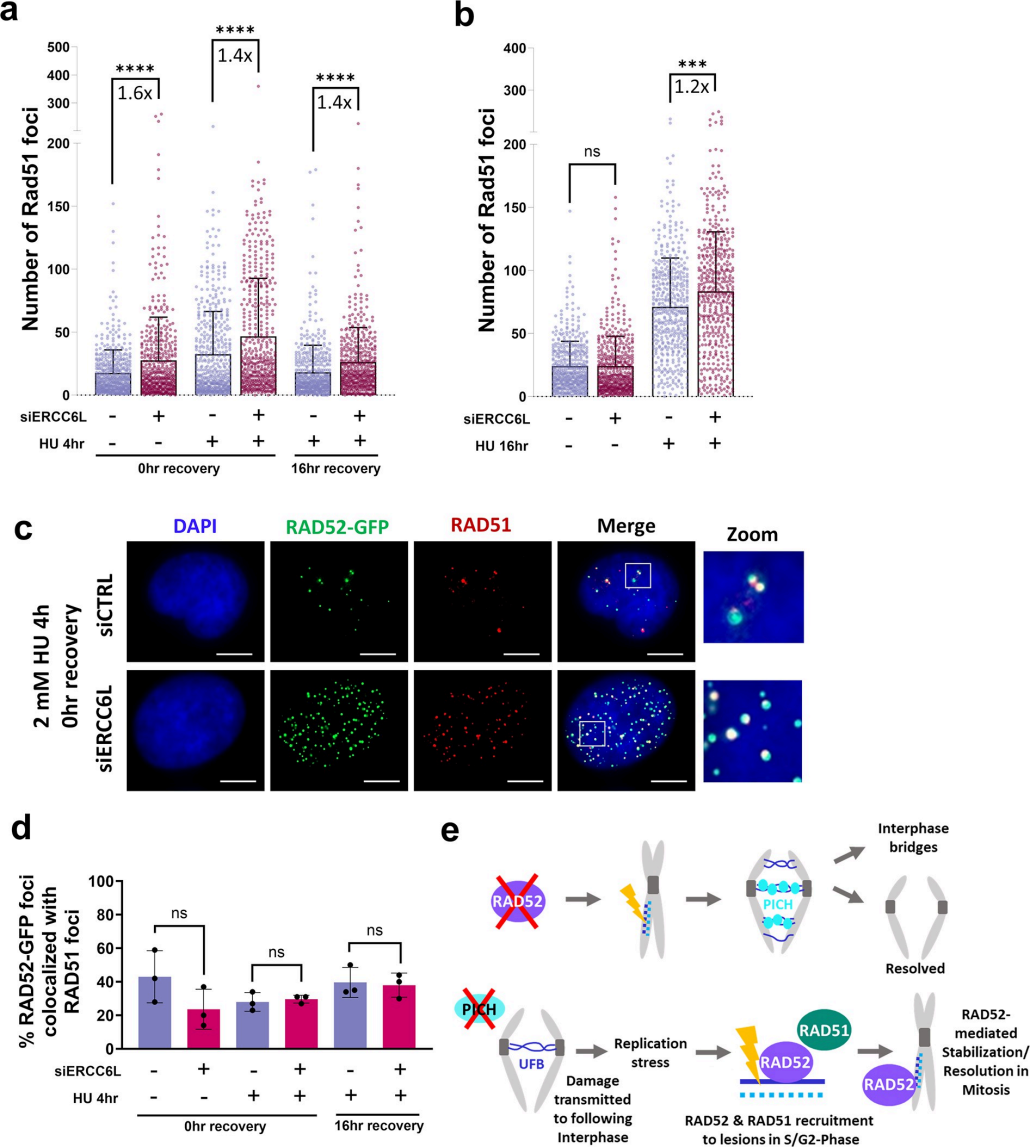
ERCC6L depletion induces RAD51 foci in interphase cells that co-localize with RAD52-GFP foci. **a-b)** RAD51 foci in interphase cells significantly increase with siERCC6L treatment (pool of 4 siRNAs) both with and without HU treatment. Experiments were performed with the same conditions shown in Fig 7A for ERCC6L depletion and HU exposure. Bars show mean foci value. The number of nuclei (N) analyzed per condition are N = 456–480 (a) and N = 457–468 (b). ns = not significant (p-value shown), ** = p<0.01, and **** = p<0.0001, K-S test. **c)** Shown are representative images of interphase cells to detect co-localization of RAD52-GFP and RAD51 foci. Scale bars are 10 μm and images were taken at 40x magnification. White boxes on merge show location of zoom. **d)** The percentage of RAD52-GFP foci that colocalize with RAD51 foci in cells with ≥10 RAD52-GFP foci is not significantly different in cells treated with siCTRL or siERCC6L with or without HU exposure. Treatment conditions are identical to those shown in (a). N = 3 independent experiments. ns = not significant, unpaired *t*-test. **e)** Speculative model of the co-compensatory relationship between RAD52 and ERCC6L to mitigate genotoxic stress.

Prior studies have shown that in addition to strand annealing capabilities, RAD52 is also involved in RAD51-mediated strand exchange [2,69,70]. Therefore, we wondered whether combining depletion of ERCC6L and HU treatment might also cause foci formation of RAD51. To test this, we treated the RAD52-GFP cell line with conditions identical to those used to investigate RAD52-GFP localization after HU exposure and ERCC6L depletion (Fig 7A) but stained and analyzed RAD51 foci by IF microscopy. We observed modest, but significant increases in RAD51 foci with siERCC6L treatment alone, and with 4-hour HU exposures both with and without recovery (Fig 8A, 1.6-fold, 1.4-fold, and 1.4-fold, respectively). We also performed a long exposure to HU (16-hours) and found that siERCC6L treatment also caused a modest increase (1.2-fold) in RAD51 foci (Fig 8B). Finally, at this longer timepoint, siERCC6L treatment without HU did not cause a significant increase in RAD51 foci. Altogether, ERCC6L depletion appears to cause an increase in RAD51 foci in interphase, but to a lesser magnitude (as indicated by fold-increase) than for RAD52 foci (Fig 7C and 7D).

We next wondered whether RAD52 and RAD51 are recruited to the same lesions in interphase in response to HU exposure and/or ERCC6L depletion. To assess this, we measured the percentage of RAD52-GFP foci that colocalize with RAD51 foci (Fig 8C). We observed modest colocalization between RAD52-GFP and RAD51 foci ranging between 25–43% across all conditions (Fig 8D). Moreover, the average percentage of colocalized foci were similar with and without siERCC6L treatment. This suggests that as RAD52-GFP and RAD51 foci increase in response to ERCC6L depletion, the number of colocalized foci increases proportionally. In total, these findings suggest that RAD51 is recruited in interphase in response to ERCC6L depletion combined with replication stress, and such foci co-localize with RAD52 at similar frequencies with each treatment.

## Discussion

To investigate factors and pathways important for cellular fitness in cells without RAD52, we performed genome-wide CRISPR knock-out screens, which revealed hundreds of factors with a differential effect on RAD52^KO^ vs. RAD52^WT^ cells. Using this list of genes, we performed pathway analysis to identify a series of factors for secondary screening from pathways related to the nucleus. From such secondary screening, we found that siRNA targeting of 5 factors in RAD52^KO^ cells induces a significant increase in G1-phase 53BP1 foci formation and negatively impacts clonogenic survival. These factors included ERCC6L (also known as PICH), DHX9, GLE1, USP1, and CDC6. Although all 5 of these factors represent interesting targets, we focused our attention on ERCC6L for the rest of the study. Our subsequent cell biology analysis indicates that these two factors co-compensate, and thus we envision the following model. In the case of RAD52 loss, we posit that unresolved lesions persist into anaphase of mitosis and cause an increase in ERCC6L-UFBs. The fate of these ERCC6L-UFBs is either subsequent resolution or persistence into the following interphase where they form chromatinized DNA bridges (Fig 8E, top). Additionally, we propose that loss of ERCC6L and subsequent failure to resolve UFBs in mitosis causes persistent lesions into the following interphase. RAD52 and RAD51 are recruited to these lesions in S- and G2-phases, while added replication stress enhances such recruitment. Some lesions remain into mitosis where RAD52 continues to stabilize them and/or promote their resolution (Fig 8E, bottom).

We observed these co-compensation effects of RAD52-GFP foci and ERCC6L-UFBs without exogenous stress, but we also examined these effects following various stress treatments. Such treatments provide insight into the types of stress that amplify the co-compensation of ERCC6L and RAD52, as well as the relative timing of these effects following the treatments. First, we examined effects of the Topo IIα inhibitor ICRF-193, which appears to cause replication stress that leads to UFBs [64]. Indeed, we found that ICRF-193 treatment induces ERCC6L-UFBs, but notably, the frequency of such UFBs was higher in the RAD52^KO^ cell line, as was also the case under unstressed conditions. We suggest that RAD52 and Topo IIα are each important to suppress replication stress that causes ERCC6L-UFBs. While ERCC6L appears to mark all UFBs, other factors are known to mark subsets of UFBs. For example, one such subset of UFBs has also been shown to stain with FANCD2 at the ends. To contrast such FANCD2-UFBs with those we found with RAD52 loss, we note that prior studies have shown that FANCD2-UFBs are not induced by topoisomerase II inhibition [32]. In contrast, RAD52 loss combined with topoisomerase II inhibition causes a marked increase in UFBs. We also posited that combined loss of RAD52 and ICRF-193 treatment may cause an increased reliance on ERCC6L. Consistent with this notion, we found that combined loss of RAD52, depletion of ERCC6L, and ICRF-193 treatment caused a marked loss of clonogenic survival. Altogether, these findings indicate that loss of RAD52 causes an increased reliance on ERCC6L both under unstressed conditions, and with inhibition of Topo IIα.

To examine RAD52-GFP foci formation with combinations of ERCC6L depletion and replication stress, we performed a series of HU treatments. These experiments provided an indication of when RAD52 recruitment occurs with respect to the timing of the source of genotoxic stress. In particular, we found that cells depleted of ERCC6L and exposed to HU (4 hours) with a long recovery time (16 hours) had dramatically increased RAD52-GFP foci in interphase (3.8-fold, Fig 7C). In contrast, with HU treatment but without a recovery time, ERCC6L depletion did not cause an increase in RAD52 interphase cells. We suggest that the specific increase in RAD52-GFP foci in interphase following ERCC6L depletion and HU treatment with a long recovery time reflects RAD52 recruitment to genotoxic stress that originated in the prior cell cycle. However, we cannot exclude that ERCC6L depletion causes increased RAD52-GFP foci in interphase without requiring the cells to cycle through mitosis. Our data also provide some insight for when RAD52 forms foci in interphase. For siERCC6L treatment alone, we found that the increase in RAD52 foci was limited to EdU-negative cells. Assuming these EdU-negative cells represent G2-phase cells, this result would indicate that such RAD52 foci form after S-phase. We cannot exclude the possibility that these EdU-negative cells could represent G1-phase cells. However, given that we also observe a marked increase in RAD52 foci in prometaphase cells with ERCC6L depletion, we suggest that ERCC6L depletion causes RAD52 foci late in the next cell cycle phase (i.e., G2-phase and prometaphase cells, **Fig 8E**). In contrast, ERCC6L depletion, combined with HU treatment and recovery, appears to also cause RAD52 foci in S phase (i.e., both EdU-positive and EdU-negative cells). This finding indicates that combining ERCC6L depletion with HU treatment and recovery cause a type of lesion in S-phase that recruits RAD52 foci, which is not caused by ERCC6L depletion alone.

As for what role RAD52 is playing in interphase in response to ERCC6L depletion, one possibility is that RAD52 is needed to protect lesions resulting from unresolved UFBs from triggering further instability in the following S-phase. Recent studies have identified protective roles for RAD52 during S-phase replication, where loss of RAD52 was associated with unscheduled fork reversal and degradation, accumulation of single stranded DNA (ssDNA), and reliance on RAD51 and origin re-priming (Polα) to recover damaged forks and mitigate further instability [23,71]. Thus, we considered that ERCC6L depletion might also cause RAD51 foci. While we found an increase in RAD51 foci after ERCC6L depletion, particularly after HU treatment, the co-localization frequencies with RAD52 foci were modest. We suggest that RAD51 and RAD52 may function together to some degree, but also appear to be playing distinct roles in resolution of replication stress caused by ERCC6L depletion and HU treatment.

Another possible role for RAD52 in interphase is to recruit and bind to other factors involved in replication fork degradation and breakage. It was previously shown that RAD52 and MUS81 cooperate to rescue regressed replication forks, particularly in the context of checkpoint deficiency (i.e., CHK1 inactivation) [72]. Moreover, in the context of BRCA-deficiency, it was shown that deprotected replication forks are susceptible to degradation by MRE11 and EXO1, and require MUS81 for POLD3-mediated restart [73]. Additionally, RAD52 was shown to protect stalled replication forks from excessive, MRE11-mediated degradation under BRCA-proficient conditions as well [23].

We also examined the role of RAD52 and ERCC6L in response to another cellular stress: G2/M arrest via RO3306 treatment. Such treatment is often used to enrich for prometaphase cells, such as for MiDAS analysis (see below), but recent studies have also indicated that RO3306 alters the cellular response to replication stress, which may reflect defects in modulating the timing of G2/M progression with DNA replication [66]. While we found that ERCC6L depletion caused elevated RAD52-GFP foci in prometaphase cells both with and without RO3306 treatment, HU treatment caused an increase in RAD52-GFP foci only if the HU treatment was followed by RO3306 treatment. These findings indicate that RO3306 treatment may cause an increased dependency on RAD52 in prometaphase cells, which we then assessed using clonogenic survival experiments. Indeed, we found that a long (48-hour) RO3306 exposure has a negative impact on clonogenic survival in RAD52^KO^ cells. Consistent with ERCC6L and RO3306 having distinct effects on the requirement for RAD52, we further found that the combination depletion of ERCC6L and RO3306 treatment caused a marked loss in viability in RAD52^KO^ cells. Based on these results, we posit that arrest in late G2-phase allows additional time for RAD52 recruitment to lesions induced by siERCC6L and HU treatment. Altogether, given the role of ERCC6L in mitosis, and that RO3306 disrupts G2/M progression, these findings support a key role of RAD52 to respond to replication stress in mitosis.

As discussed above, our data show that RAD52-GFP forms foci in prometaphase in response to ERCC6L depletion. Initially we hypothesized that such RAD52-GFP foci were sites of mitotic DNA synthesis, since RAD52 has been implicated in such mitotic DNA synthesis (i.e., MiDAS) [13–18]. However, a recent study found that RAD52 is dispensable for MiDAS induced by APH in RPE-1 cells [74]. Similarly, our findings also indicate that the role of RAD52 in mitosis is not necessarily linked to MiDAS, Namely, we found that RAD52-GFP foci do not often localize with EdU incorporation, and siERCC6L treatment causes an increase in RAD52-GFP foci without causing an increase in EdU foci. Thus, we propose that RAD52 may be playing a structural role in prometaphase cells to stabilize and resolve lesions related to replication stress rather than mediating DNA synthesis per se.

Notably, previous studies have identified BRCA2 as showing synthetic lethality with RAD52 [29]. Although BRCA2 was not identified in our primary screen, we nevertheless did observe a decrease in clonogenic survival of RAD52^KO^ cells with BRCA2 siRNA depletion in our RPE-1 cell lines used for the genome-wide screens (Fig 3F). Upon inspection of the beta scores produced by our primary genome-wide screens, we noted that neither IR nor Cis-Pt exposure had any effect on fitness of cells that received BRCA2 guides (panel (c) in S4 Fig), despite evidence of hypersensitivity to IR and Cis-Pt in BRCA2 deficient cells [75–79]. We speculate that the sgRNAs targeting BRCA2 are not effective under these screening conditions. While there are many possible explanations for why sgRNAs may fail to efficiently knock-out a given target gene, some documented possibilities include a high efficiency of error-free repair of Cas9-induced DSBs, and sequence contexts that favour microhomology-mediated deletions that remain in the reading frame [80,81]. In contrast, beta scores for other factors in the FA, ATM, and NHEJ pathways exhibited the expected loss of fitness in the screens with IR and Cis-Pt exposures (Fig 1D). Furthermore, beta scores for the RAD51 paralogs RAD51B and RAD51D (previously reported to be synthetic lethal with RAD52 loss [30]) indicate a loss of fitness in the RAD52^KO^ line as compared to RAD52^WT^ under at least some of the screening conditions (panel (c) in S4 Fig). Further, two known RAD52 synthetic lethal interactors, RAD51D [30] and XAB2 [82], were identified as hits in our genome-wide screens, though they were not selected as hits in our secondary screen based on the G1 53BP1 foci assay (S4 Table and S2 Fig).

A recent study demonstrated a variety of factors that are synthetic lethal with ERCC6L via genome-wide loss of function screens [83]. These screens in HAP1 cells, a near-haploid leukemia-derived cell line, did not identify RAD52 as a synthetic lethal interactor. A number of factors may have influenced the lack of reciprocal results with our screen here. For one, as a haploid line, the altered number of chromosomes may lead to differences in mitosis and genetic dependencies on factors important for chromosomal segregation (i.e., ERCC6L) compared to diploid lines. Additionally, in contrast to the p53^KO^ RPE-1 cell lines used in our studies, p53 is at least partially functional in HAP1 cells [84], and thus may also influence how these cells mitigate genotoxic stress.

Regarding potential clinical significance of our findings, recent work has shown that ERCC6L is often over-expressed in breast cancers and that knockdown of ERCC6L inhibits proliferation in breast cancer cells [85]. Similarly, loss of ERCC6L in triple negative breast cancer causes chromosome instability and cell death [86]. More broadly, pan-cancer studies of altered ERCC6L expression have identified increased ERCC6L expression as a marker of poor prognosis in multiple types of cancer [87]. Finally, RAD52 inhibitors are in pre-clinical development [27]. Thus, we suggest that evaluating ERCC6L levels and mutations in cancer should be investigated as a potential biomarker for response to targeting RAD52, and vice versa.

## Materials & methods

### Cell lines and plasmids

The parental cell line used in these studies (referred to as RAD52^WT^) is a line of hTERT RPE-1 human epithelial cells that are p53^KO^ and stably express Cas9-FLAG that was generously provided by Dr. Daniel Durocher (Lunenfeld-Tanenbaum Research Institute, University of Toronto), and cultured as described [44,45]. The RAD52^KO^ cell line was generated from the RAD52^WT^ line by CRISPR-Cas9-mediated deletion of the 1.1kb region between exons 3 and 4 using guide RNAs sg1 and sg2 cloned into px330 (Addgene #42230) as previously described [88]. These sgRNA vectors were co-transfected with a dsRED expression vector using ViaFect transfection reagent (Promega #E4981). After 3 days, cells were sorted to enrich dsRED-positive cells (BD Aria) and plated at low density to achieve single colonies. The RAD52^KO^ cell line was then isolated through screening individual clones by PCR (oligos ol1 and ol2) and Sanger sequencing. Knock-out of the RAD52 protein was confirmed by Immunoblot analysis. All sgRNAs and oligos are listed in (S1 Table).

The RAD52^KO^ U2OS and RAD52^WT^ U2OS lines were previously described (in [88] and [89] respectively). The RAD52^KO^ H2B-GFP and RAD52^WT^ H2B-GFP cell lines were generated by transfecting the RAD52^KO^ and RAD52^WT^ RPE-1 cell lines with 400 ng of an H2B expression vector tagged with EGFP at the C-terminus (Addgene #11680) [90]. The transfection was performed in a 6-well plate with 2ml of antibiotic free media and using Viafect transfection reagent. Cells were subsequently grown in media containing 600 μg/ml G418 (Corning #61-234-RG) for 12 days and then sorted for GFP-positive cells.

The RAD52-GFP cell line was generated by transfecting the RAD52^WT^ cell line with 800 ng of a RAD52 expression vector that is tagged with GFP at the N-terminus that was generously provided by Dr. Markus Lobrich (Technical University of Darmstadt) [65]. The transfection was performed in a 6-well plate with 2 mL of antibiotic free media and using Viafect transfection reagent. There was no antibiotic selection used, but instead cells were sorted to enrich for GFP-positive cells, which were cultured and sorted a second time (BD Aria). At the start of each experiment, cells were analyzed for the GFP-positive percentage by flow cytometry (Quanteon) and only used if GFP-positive cells exceeded 50% of the total population. Cells were also periodically re-sorted to maintain RAD52-GFP expression. All cell lines used in experiments tested negative for mycoplasma contamination (Lonza MycoAlert PLUS Mycoplasma Detection Kit).

### CRISPR-KO screens

CRISPR-KO screens were carried out as described [44], but with some modifications. Specifically, RAD52^KO^ and RAD52^WT^ cells were transduced at a low multiplicity of infection (MOI<0.3) with lentivirus containing the BFP-tagged Human Improved Genome-wide Knockout CRIPSR Library (Addgene #67989) [91] packaged into lentivirus (via 293T cells). MOI was confirmed for both cell lines two days after transduction by flow cytometry analysis (Quanteon) where MOI was equivalent to the percentage of BFP-positive cells. Following transduction, cells were treated with media containing 20 μg/ml puromycin to select for and enrich cells transduced with the library. After 24 hours, cells were trypsinized and resuspended in fresh media with 20 μg/ml puromycin and incubated for another 24 hours. At 2 days post-transduction (considered to be the initial time point, T0), puromycin was removed. At 8 days post-transduction (T6), the total cells were split into 3 different treatment groups: Untreated, 2 Gy Ionizing Radiation exposure (IR), and 1 μM cisplatin treatment (Cis-Pt). Cells in the IR screen were irradiated once (Gammacell 3000) on T6, while cells in the Cis-Pt screen were grown in media containing 1 μM cisplatin for 6 days (T6-T12). Cells were passaged to maintain 400x coverage of the CRISPR library every 3 days through the duration of the screening period (T3-T18).

Cell pellets were collected from T0 and T18 time points for analysis, where the number of cells in each pellet (~36 million cells) was set to represent the total number of guides in the CRISPR library (90,709 guides) multiplied by the desired library coverage for sequencing (400x). Cell pellets were processed into genomic DNA using the QIAmp Blood Maxi Kit (Qiagen #51192), and the integrated sgRNA library was amplified from the genomic DNA by PCR using Q5 Hot Start High-Fidelity 2x Master Mix (NEB #M0494L) and oligos ol3 and ol4 (S1 Table). Amplified and gel purified PCR products were sequenced by Illumina HiSeq (Azenta), and the resulting reads were analyzed using the maximum-likelihood estimation (MLE) algorithm from the MAGeCK pipeline [49] to generate sgRNA counts and selectivity (beta) scores (by comparing guide counts from the T18 to the T0 timepoint for each gene represented in the library) for comparing the RAD52^WT^ and RAD52^KO^ cell lines in each of the three screens.

### Gene hit selection for secondary screening

The MAGeCKFlute pipeline [50] was used to perform downstream analyses for results from MAGeCK MLE results, described above. First, this pipeline includes beta score normalization, which uses each sample’s beta scores and a list of common essential genes to normalize all beta scores between samples to account for cell division rate differences [50]. Second, we used MAGeCKFLute to determine gene hits by applying standard deviation cutoffs to plotted normalized beta scores of 1.5x the standard deviation to the x, y, and diagonal axes. Gene “hits” were selected as those in the section of the plot that were within the x-axis cutoffs (RAD52^WT^ beta score 1.5x standard deviation from x = 0), but beyond the negative y-axis and diagonal cutoffs (RAD52^KO^ beta score <1.5x standard deviation from y = 0 and diagonal) (see Fig 1C and S2 Table). Gene set enrichment analysis (GSEA) was then performed on the hits selected from each individual screen with MAGeCKFlute using default parameters (S3 Table). GSEA enriched ontologies with FDR <0.01 and NES scores of >0.5 from all three screen conditions were combined (for a total of 118 enriched ontologies) and categorized into 6 groups based on the ontology names. These groups included (1) DNA damage and repair, (2) Cell cycle control and cell division, (3) Nuclear RNA metabolism, (4) Translation and Mitochondrial RNA metabolism, (5) Response to stimuli and signaling, and (6) Protein modification and metabolism. The full lists of ontologies that make up each of these summary categories, and which screen the enriched genes were identified in (untreated, IR or Cis-Pt treated) are detailed in (S3 Table). We then focused on the first three groups, which involved processes that function inside the nucleus (IN) vs. those function outside the nucleus (ON). To further narrow down the screen hits identified by GSEA for secondary screening, we selected 59 gene hits from those enriched IN pathways based on the following criteria: at least one gene from each enriched ontology was selected, and genes that were reported as hits in multiple ontologies, multiple screen conditions, or multiple components of the same complex were favored. We also favored enriched screen hits with known related functions or interactions with RAD52, (i.e., RAD51D and RPA1).

### Immunoblot analysis

Cells were lysed with either NETN buffer (20  mM TRIS (pH 8.0), 100 mM NaCl, 1 mM ethylenediaminetetraacetic acid (EDTA), 0.5% IGEPAL, 1.25  mM dithiothreitol and Roche Protease Inhibitor) and a series of freeze-thaw cycles (RAD52 blot), or ELB buffer (250 mM NaCI, 5 mM EDTA, 50 mM HEPES, 0.1% Ipegal, Roche protease inhibitor) with sonication (Qsonica, #Q800R) (Cas9-FLAG, ERCC6L, DHX9, GLE1, USP1, CDC6, BLM, and BRCA2 blots). Blots were probed using antibodies against RAD52 1:500 (Santa Cruz Biotechnology #sc365341), FLAG 1:1000 (Sigma #A8592), ERCC6L 1:500 (Abnova #H00054821-D01P), DHX9 1:250 (BETHYL #A300-855A), GLE1 1:500 (Abcam #ab96007), USP1 1:1000 (BETHYL #A301-698A), CDC6 1:1000 (Santa Cruz #sc56273), BLM 1:1000 (BETHYL #A300-110A) BRCA2 1:1000 (EDM Millipore #OP95), or ACTIN 1:1000 (Sigma #A2066), and with HRP-conjugated secondary antibodies: rabbit anti-mouse 1:3000 (Abcam #ab205719) or goat anti-rabbit 1:3000 (Abcam #ab205718). ECL Immunoblotting Substrate (Thermo Fisher Scientific #32106) was used to detect HRP signal on film. Immunoblot quantification was performed using Fiji (ImageJ), and band intensity was reported relative to Actin intensity (loading control).

### Cell biology assays

For the G1 53BP1 foci and micronuclei assays with siRNA knockdown, RAD52^KO^ and RAD52^WT^ cells were seeded at 5 x 10^4^ cells per well in 12-well plates with 1 mL of antibiotic-free media and treated with Lipofectamine RNAiMAX (ThermoFisher #13778075) and 10 pmol of siRNA pools (pool of 4 siRNAs; Dharmacon siGENOME, sequences from manufacturer in S1 Table) or non-targeting siRNA (siCTRL; Dharmacon #D-001810-01-20). After 16–18 hours, cells were trypsinized, resuspended, and transferred to coverslips in 6-well plates. 3 Days after siRNA treatment, cells were fixed in 4% paraformaldehyde (PFA), permeabilized with 0.5% Triton X-100 in PBS, and blocked with 8% goat serum in PBS. For the G1 53BP1 assay, fixed cells were incubated with rabbit 53BP1 antibody 1:500 (Abcam #ab36823) and mouse Cyclin A antibody 1:100 (Santa Cruz #sc-271682). Secondary antibodies (goat-anti-rabbit 488 (Invitrogen #A-32731) and goat-anti-mouse 594 (Invitrogen #A-11032) were applied at 1:500. For the micronuclei assay, cells were only mounted with VECTASHIELD antifade mounting medium with DAPI (Vector labs #H-1200-10).

For the G1 53BP1 foci assay with CRISPR-Cas9-mediated sgRNA knockdown, RAD52^KO^ and RAD52^WT^ cells were seeded at 2.5 x 10^4^ cells per well in a 24-well plate with 0.5 mL of media (with antibiotics). Lipofectamine Cas9 Plus (Thermofisher #CMAX00008), 1.95 pmol sgRNA mix (combined sg3, sg4, and sg5 –sequences from manufacturer listed in S1 Table) or sgCTRL (Synthego Negative Control sgRNA (mod) #1), and 10 pmol Cas9 (Synthego Cas9 nuclease 2NLS, S. pyrogenes) were combined and added to cells with Lipofectamine CRISPRMax (Thermofisher #CMAX00008). After 24 hours, cells were trypsinized and transferred to 6-wells and grown for 5 days, passaging as needed. 6 days after the transfection, cells were trypsinized, resuspended and transferred to coverslips in 6-well plates at a density of 1 x 10^5^ cells per well. 8 days after the transfection, cells were fixed and stained using the same method as performed for the G1 53BP1 foci assay with siRNA knockdown.

For the Anaphase ultra-fine bridge and chromatin bridge assays, RAD52^KO^ and RAD52^WT^ cells were seeded at 1 x 10^5^ cells per well in 6-well plates with 2 mL of antibiotic-free media along with Lipofectamine RNAiMAX and 20 pmol of siCTRL. After 16–18 hours, cells were trypsinized, resuspended, and transferred to coverslips in 6-well plates. For experiments where cells were treated with ICRF-193 (Enzo Life Sciences #BML-GR332-0001), 3 days after transfection, 200 nM ICRF-193 or equivalent volume of vehicle (DMSO) was added to each well for 6 hours prior to fixation. Afterwards, cells were washed and allowed to recover for 16 hours prior to fixation. For all experiments, cells were pre-extracted with buffer A (0.2% Triton X-100 in 1x PEM buffer: 10x PEM buffer: 200 mM HEPES pH 7.2–7.4, 10 mM MgCl_2_, 100 mM EGTA, diluted to 1x in PBS), and fixed in buffer B (0.1% Triton X-100, 8% PFA in 1x PEM buffer), which is similar to previously described [92]. Fixed cells were blocked in PGST (8% goat serum, 0.5% Triton-X-100 in PBS) for at least 24 hours at 4°C. Cells were then incubated at 4°C overnight with rabbit ERCC6L antibody (Abnova #H00054821-D01P, diluted 1:100) prepared on ice. Cells were then incubated with goat-anti-rabbit 488 (Invitrogen) secondary antibody.

For mitotic RAD52-GFP foci experiments, RAD52-GFP-expressing cells were seeded at 1 x 10^5^ cells per well in 6-well plates with 2 mL of antibiotic-free media along with Lipofectamine RNAiMAX and 20 pmol of either a pool of 4 siRNAs targeting ERCC6L (siERCC6L; Dharmacon #M-031581-01-0005, sequences from manufacturer in S1 Table) or siCTRL. After 16–18 hours, cells were trypsinized, resuspended, and transferred to coverslips in 6-well plates. For experiments assaying prometaphase cells, 2 days after transfection, cells were exposed to 2 mM HU or equivalent volume of DMSO for 4 hours, washed, and allowed to recover in fresh media for 12 hours. In experiments where cells were synchronized in G2/M and released into mitosis prior to fixation, 7 μM RO3306 (CDK1 inhibitor; Selleck chemicals #S7747) was added and cells were incubated for 6 hours. Cells were then washed, incubated for 30 minutes in fresh media, and fixed. In experiments where cells were not synchronized, cells were allowed to recover from HU exposure for 18 hours in fresh media and then fixed. For experiments where ERCC6L was knocked down using CRISPR-Cas9 sgRNA, cells were seeded at 2.5 x 10^4^ cells per well in a 24-well plate and transfected using the same method as the G1 53BP1 foci assay with sgRNA ERCC6L knockdown. 24 hours after transfection, the cells were trypsinized and transferred to 6-wells and grown for three days, passaging as needed. Four days after the transfection, cells were trypsinized, resuspended, and transferred to coverslips in 6-well plates at a density of 1 x 10^5^ cells per well. Six days after the transfection, cells were exposed to 7 μM RO3306 and incubated for 6 hours to synchronize cells in G2/M. Cells were then washed to release the cells into mitosis, incubated for 30 minutes in fresh media, and fixed.

For experiments assaying RAD52-GFP foci in interphase cells with a 4-hour HU exposure, cells were seeded similarly to mitotic experiments and 2 mM HU or equivalent volume of DMSO was added 2 days after transfection and incubated for 4 hours. Experiments without recovery time were fixed immediately after the 4-hour exposure, while experiments with recovery were washed and allowed to recover for 16 hours in fresh media prior to fixation. For experiments using these conditions that also included labeling with 5-ethynyl-2’-deoxyuridine (EdU), cells were pulsed with 10μM EdU in media for 1 hour prior to fixation. For experiments with 16-hour HU exposure, 2 mM HU or equivalent volume of DMSO was added to cells 2 days after transfection and cells were incubated for 16 hours followed by fixation. For both mitotic and interphase RAD52-GFP experiments, cells were fixed by the same method used for anaphase ultrafine bridge assay, which is similar to the method previously described in [92].

In experiments where interphase cells were analyzed, fixed cells were blocked with PGST for at least 24 hours at 4°C. To evaluate cell cycle profiles, one set of experiments was also incubated with mouse Cyclin A antibody (1:100) for 1 hour, followed by goat-anti-mouse 594 secondary antibody (1:500) for 30 minutes. Experiments that were repeated with an EdU pulse before fixation were processed using the EdU Click-iT detection kit (Thermofisher #C10639). For experiments in interphase cells that were stained to evaluate RAD51 foci, cells were incubated with Rabbit RAD51 antibody (Calbiochem # PC130, diluted 1:500) for 2 hours, followed by goat-anti-mouse 594 secondary antibody (1:500) for 30 mintues.

For the MiDAS assay, RAD52-GFP-expressing cells were seeded and transfected with siRNA similarly to RAD52-GFP foci experiments. For experiments with Aphidicolin (APH) treatment, cells were treated with 0.4 μM APH or equivalent volume of DMSO (added 2 days after transfection) for a total of 46 hours. For experiments with HU treatment, 2 mM HU or equivalent volume of DMSO was added 3 days after transfection, and cells were incubated for 4 hours. HU-treated cells were washed and allowed to recover for 12 hours. Both HU- and APH-treated cells (and DMSO-treated controls) were then synchronized by 7 μM RO3306 treatment for 6 hours. Cells were washed and released into mitosis in media containing 20 μM EdU and incubated for 30 minutes. Cells were fixed using the same method used for anaphase ultrafine bridge assay, which is similar to previously described [92]. Fixed cells were processed using the EdU Click-iT detection kit (Thermofisher #C10639), but with modified reaction components as previously described [68].

For mounting, imaging acquisition, and image analysis, coverslips from all cell biology experiments were mounted with VECTASHIELD antifade mounting medium with DAPI (Vector labs #H-1200-10) prior to imaging. Images from the anaphase ultra-fine bridge assay experiments were acquired using an Olympus IX71 microscope with a 60x oil objective. Images from all other cell biology experiments were acquired using a ZEISS Axio Observer II microscope with a 20x air objective (G1 53BP1 assay and chromatin bridge assay) or 40x oil objective (Micronuclei, RAD52-GFP and RAD51 foci, and MiDAS assays). Foci (53BP1, RAD52-GFP, RAD51, and mitotic EdU) and Cyclin A or interphase EdU intensity were quantified using Fiji (ImageJ). Micronuclei, anaphase ERCC6L ultra-fine bridges (UFBs), and chromatin bridges were quantified manually. All foci, anaphase UFBs, micronuclei and chromatin bridges were counted from at least 3 independent coverslips for each condition and plated on at least 2 different days, except for the siRNA subscreen that was performed with one set of coverslips per gene. Colocalization analysis (RAD52-GFP with EdU and RAD52-GFP with RAD51) was performed using an automated ImageJ macro to determine XY positions of foci maxima. Distances between foci were then calculated using the following formula where x and y are the X and Y coordinates for focus 1 vs. focus 2:

$$\sqrt{\left[{\left(x_{2}-x_{1}\right)}^{2}+{\left(y_{2}-y_{1}\right)}^{2}\right]}$$

Foci were defined as colocalized if the distance between the two foci maxima was less than 3 pixels (~0.34 μm). The ImageJ macro and excel sheet used for comparison of foci maxima can be downloaded from https://github.com/baosia/FociXY.

### Live cell imaging

RAD52^KO^ H2B-GFP and RAD52^WT^ H2B-GFP cells were seeded at 1 x 10^5^ cells per well in 6-well plates and grown for 72 hours before imaging on a ZEISS Axio Observer Z1 live-cell fluorescence microscopy system. Imaging duration was 12 hours with photos (frames) taken every 2 minutes with a 20x magnification air objective. Imaging was analyzed using the ZEN lite v3.1 software to count frames to determine the duration of mitosis in mitotic cells.

### Clonogenic survival assays

For experiments with siRNA knockdown, RAD52^KO^ and RAD52^WT^ cells were seeded on day 1 at 3 x 10^4^ cells per well in 12-well plates with 1 mL of antibiotic-free media and treated with Lipofectamine RNAiMAX and 10 pmol of siRNA pools (S1 Table), or siCTRL. After 16–18 hours (on day 2), cells were trypsinized, resuspended, and re-seeded at low-density to allow the formation of colonies in 6-well plates. For drug-treated experiments, ICRF-193, RO3306, or equivalent volumes of DMSO were added to cells on day 3 and washed from cells 55 hours (ICRF-193) or 48 hours (RO3306) later.

For experiments with CRISPR-Cas9-mediated sgRNA knockdown, RAD52^KO^ and RAD52^WT^ cells were seeded and transfected similarly to the method described for the G1 53BP1 assay. 24 hours after the transfection, cells were trypsinized and transferred to 6-well plates and grown for 3 days. 4 days after the transfection, cells were trypsinized, resuspended, and re-seeded at low-density to allow the formation of colonies in 6-well plates.

For all experiments, cells were fixed 10 days after low-density seeding in 10% formalin, then stained with 0.5% crystal violet in 25% methanol, and counted using a 10x objective. Clonogenic survival frequencies were calculated by normalizing the number of colonies counted per well to the number of cells plated (plating efficiency) and then normalizing this value from each well to the mean of the parallel siCTRL/vehicle treated wells (siCTRL/DMSO = 1) (S5 Table). Each clonogenic survival value is derived from an independent well for a total of N = 6, with wells plated over at least 2 different days, except for siRNAs that showed no significant viability loss in the RAD52^KO^ vs. RAD62^WT^ line, for which only N = 3 wells were tested (panel (a) in S3 Fig).

### HR reporter assay

The DR-GFP reporter assay was performed in U2OS cells as previously described in [62]. In brief, cells were seeded at a density of 1 x 10^5^ cells in 12-well plates with 1 mL of antibiotic-free media and treated with Lipofectamine RNAiMAX and 10 pmol of siBRCA2 pool (S1 Table), or siCTRL. Cells were incubated overnight and then transfected with 200 ng of the DR-GFP-targeting sgRNA/Cas9 plasmid (previously described in [93]) using 3.6 μL of Lipofectamine 2000 in 1 mL of antibiotic-free media. Three days following the transfection, cells were analyzed by flow cytometry using a NovoCyte Quanteon Flow Cytometer System (Agilent). Transfection efficiency was also determined by transfecting cells under parallel treatment conditions with 200ng of a GFP expression vector (pCAGGS-NZE-GFP) and 200 ng of an empty vector (EV) plasmid.

## Supporting information

S1 Fig Read coverage and mapping ratios of sequencing data analyzed for each genome-wide screen condition. **a)** Sequencing achieved >30 million reads per screening condition with a mapping efficiency of >85% for all conditions. T+0 refers to the initial time point (T0), T+18 refers to samples from the final time point (T18). Treatment conditions are as follows: IR = 2 Gy Ionizing Radiation, NT = Untreated, Pt = 1μM Cisplatin.
(TIF)

S2 Fig Summary of sub-screen results for 60 genes assessed for 53BP1 foci levels. siRNA sub-screen hits from IN pathway groups 1–3 (59 genes + BRCA2, see Fig 1D) that have a significant (p<0.05 by Kolmogorov-Smirnov test) and >1.5-fold mean increase in G1 53BP1 foci in the RAD52^WT^ cell line **(a)**, G1 53BP1 foci in the RAD52^KO^ cell line **(b)**, S/G2 53BP1 foci in the RAD52^WT^ cell line **(c)**, and S/G2 53BP1 foci in the RAD52^KO^ cell line **(d)** are shown in red. For all siRNAs shown, N>50 nuclei were analyzed in both RAD52^KO^ and RAD52^WT^ lines. All siRNAs used are pools of 4 siRNAs per gene.
(TIF)

S3 Fig Impact of depletion of sub-screen hits on clonogenic survival. **a)** Clonogenic survival assay results for all 16 hits from the sub-screen (G1 53BP1 foci counts) and BLM. Colony count data is shown for RAD52^KO^ vs RAD52^WT^ lines with depletion of gene bys siRNA (pools of 4 siRNAs per gene) or siCTRL treatment and are normalized to respective siCTRL treated lines (siCTRL = 1). Statistical significance is determined by unpaired t-test, where ns = not significant, * = p<0.05, ** = p<0.01, *** = p<0.001, **** = p<0.0001. The number (N) of replicates is listed below each set of bars and reflects the number for both the RAD52^KO^ and RAD52^WT^ lines. **b)** Immunoblots confirming knock-down of genes that caused a significant viability defect in the RAD52^KO^ cell line as compared to the RAD52^WT^ line. Shown are ERCC6L, CDC6, GLE1, DHX9, and USP1 depletion via siERCC6L, siCDC6, siGLE1, siDHX9, and siUSP1 respectively in RAD52^KO^ and RAD52^WT^ cell lines. *non-specific band. **c)** Heatmap of normalized beta scores from the genome-wide screens (Fig 1B) for BLM. **d)** Immunoblots confirming knock-down of BLM by siBLM in the RAD52^KO^ and RAD52^WT^ cell lines. *non-specific bands.
(TIF)

S4 Fig Confirmation of ERCC6L expression in RAD52^KO^ and RAD52^WT^ lines, siBRCA2 reagent, and genome-wide screen fitness scores of known RAD52 synthetic lethal interactors. a) No significant difference in ERCC6L expression between RAD52^KO^ and RAD52^WT^ RPE-1 lines. (Left) Immunoblot analysis of RAD52^KO^ and RAD52^WT^ RPE-1 lines, each grown in 3 independent wells, to detect ERCC6L expression. (Right) Quantification of Immunoblot analysis. ns = not significant by *t*-test. b) Significant reduction in homology driven repair (HDR) efficiency after siBRCA2 treatment in U2OS cells. (Left) Immunoblot confirming BRCA2 depletion after siBRCA2 treatment in U2OS cells. (Right) Frequency of GFP-positive cells generated by the DR-GFP reporter (indicating repair by HDR) after siBRCA2 (pool of 4 siRNAs) or siCTRL treatment. N = 3 independent replicates. **** = p<0.0001 by *t*-test. c) Heatmap of normalized beta scores from the genome-wide screens (Fig 1B) for genes known to have synthetic lethal interactions with RAD52.
(TIF)

S5 Fig Metaphase duration, but not prometaphase or anaphase durations, are impacted by loss of RAD52. **a)** Representative examples of live cell imaging of mitotic cells in RAD52^WT^ and RAD52^KO^ cell lines. Stages of mitosis are defined below each example for the duration of the stage, except telophase (onset, identified by decondensation of chromatin). Cells were labeled with H2B-GFP and imaged at a rate of 2 min per frame. Scale bar is 20 μm, and images were taken at 20x magnification. **b)** Quantification of average phase duration for each cell line. Bars show average duration across N = 3 experiments, where >50 cells were analyzed from at least 2 fields of view over 12 hours per experiment. Significance determined by unpaired *t*-test. * = p<0.05, ns = not significant.
(TIF)

S6 Fig Experiment schematic for examining RAD52-GFP foci after treatment with sgERCC6L. **a)** Schematic of treatment with sgRNAs (pool of 3) targeting ERCC6L used to examine RAD52-GFP foci in prometaphase cells.
(TIF)

S7 Fig HU does not induce EdU foci in both siERCC6L- and siCTRL-treated cells. **a)** Schematic of treatments used for MiDAS detection in RAD52-GFP-expressing prometaphase cells exposed to either APH or HU. **b)** EdU foci increase upon APH exposure but not with HU exposure and recovery, irrespective of siERCC6L treatment (pool of 4 siRNAs). Bars show mean foci value. Significance determined by K-S test where ns = not significant and **** = p<0.0001. The number of nuclei (N) analyzed per condition are N = 157–162. **c)** The percentage of RAD52-GFP foci that colocalize with EdU foci in cells that have ≥5 EdU foci is not significantly different in cells treated with siCTRL or siERCC6L after APH exposure. Analysis of the data shown in (Fig 6C and 6D). N = 3 independent replicates. ns = not significant, unpaired *t*-test.
(TIF)

S8 Fig ERCC6L-depletion does not alter the percentage of Cyclin A-positive cells. **a)** Example IF images of interphase cells with Cyclin A co-staining (RAD52 foci are shown in Fig 7B). Treatments with siERCC6L (pool of 4 siRNAs) or siCTRL and HU are as shown in Fig 7A. Scale bars are 10 μm and images were taken at 40x magnification. **b)** siERCC6L treatment does not substantially alter Cyclin A positive levels in interphase cells. HU treatment durations and recovery times are as shown in (Fig 7A, 7C and 7D). **c)** RAD52-GFP foci in interphase cells significantly increase with siERCC6L treatment (pool of 4 siRNAs) with and without HU treatment and recovery in experiments performed with EdU incorporation. Experiments performed as shown in (Fig 7E). Bars show mean foci value. The number of nuclei (N) analyzed per condition are N = 330–342. ns = not significant, *** = p<0.001, and **** = p<0.0001, K-S test. **d)** Representative images showing RAD52-GFP foci and EdU labeling in cells treated with siERCC6L and HU. Scale bars are 10 μm and images were taken at 40x magnification.
(TIF)

S1 Table Summary of cell lines, primers, sgRNAs, and siRNAs used in this study. (XLSX)

S2 Table Genome-wide CRISPR-KO screen result statistics and selected synthetic lethal hits. (XLSX)

S3 Table Gene set enrichment analysis (GSEA), filtering, and grouping of enriched pathways. (XLSX)

S4 Table Summary of 53BP1 secondary screen. (XLSX)

S5 Table Colony counts and normalization calculations for clonogenic survival assay data. (XLSX)
