# Supplementary figures and images for: RAD52 and ERCC6L/PICH have a compensatory relationship for genome stability in mitosis
Source: PLoS Genet. 2024 Nov 19;20(11):e1011479. doi: 10.1371/journal.pgen.1011479 (PMC11614213; doi:10.1371/journal.pgen.1011479)

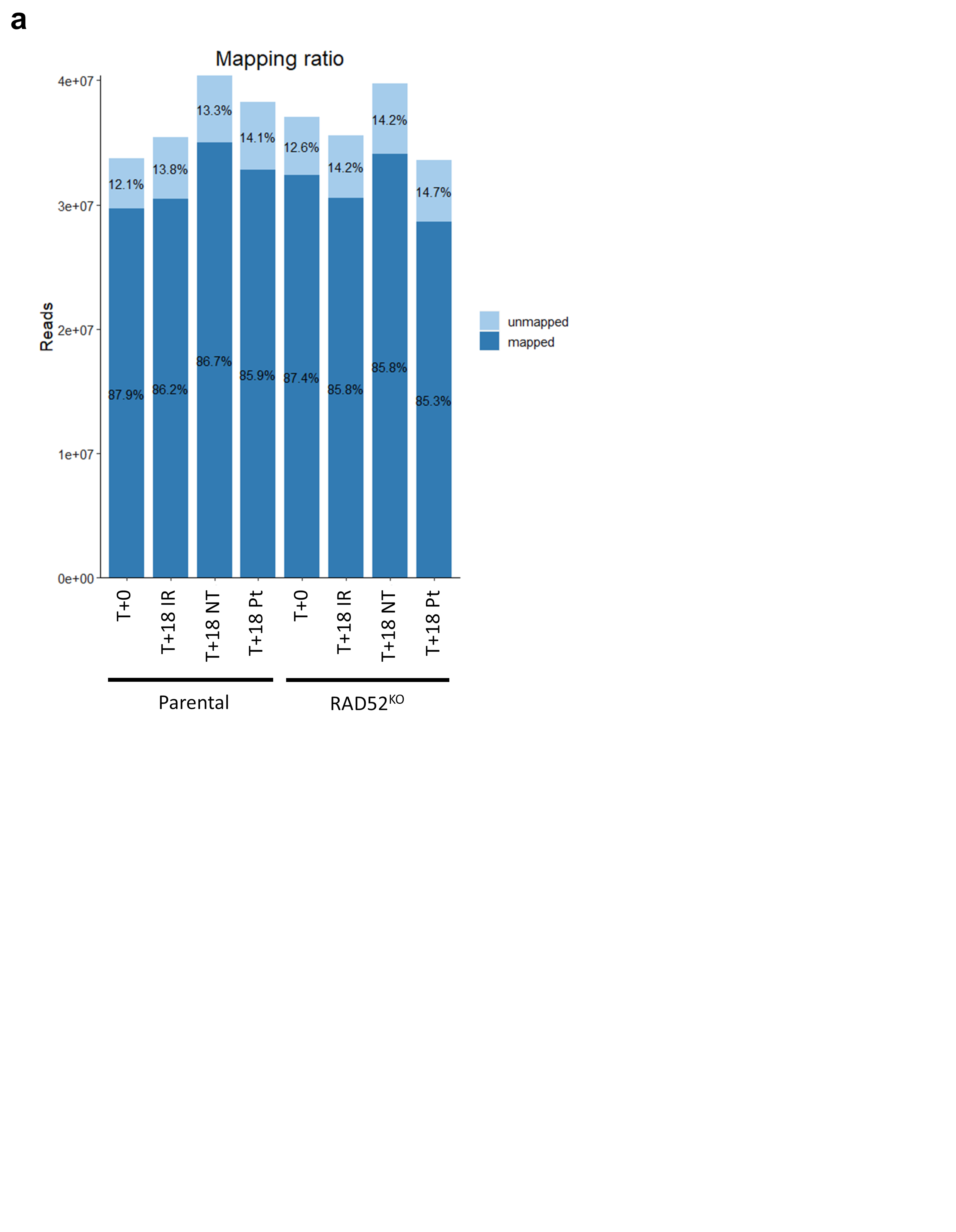

Supplement: S1 Fig — a) Sequencing achieved >30 million reads per screening condition with a mapping efficiency of >85% for all conditions. T+0 refers to the initial time point (T0), T+18 refers to samples from the final time point (T18). Treatment conditions are as follows: IR = 2 Gy Ionizing Radiation, NT = Untreated, Pt = 1μM Cisplatin. (TIF) [file pgen.1011479.s001.tif]

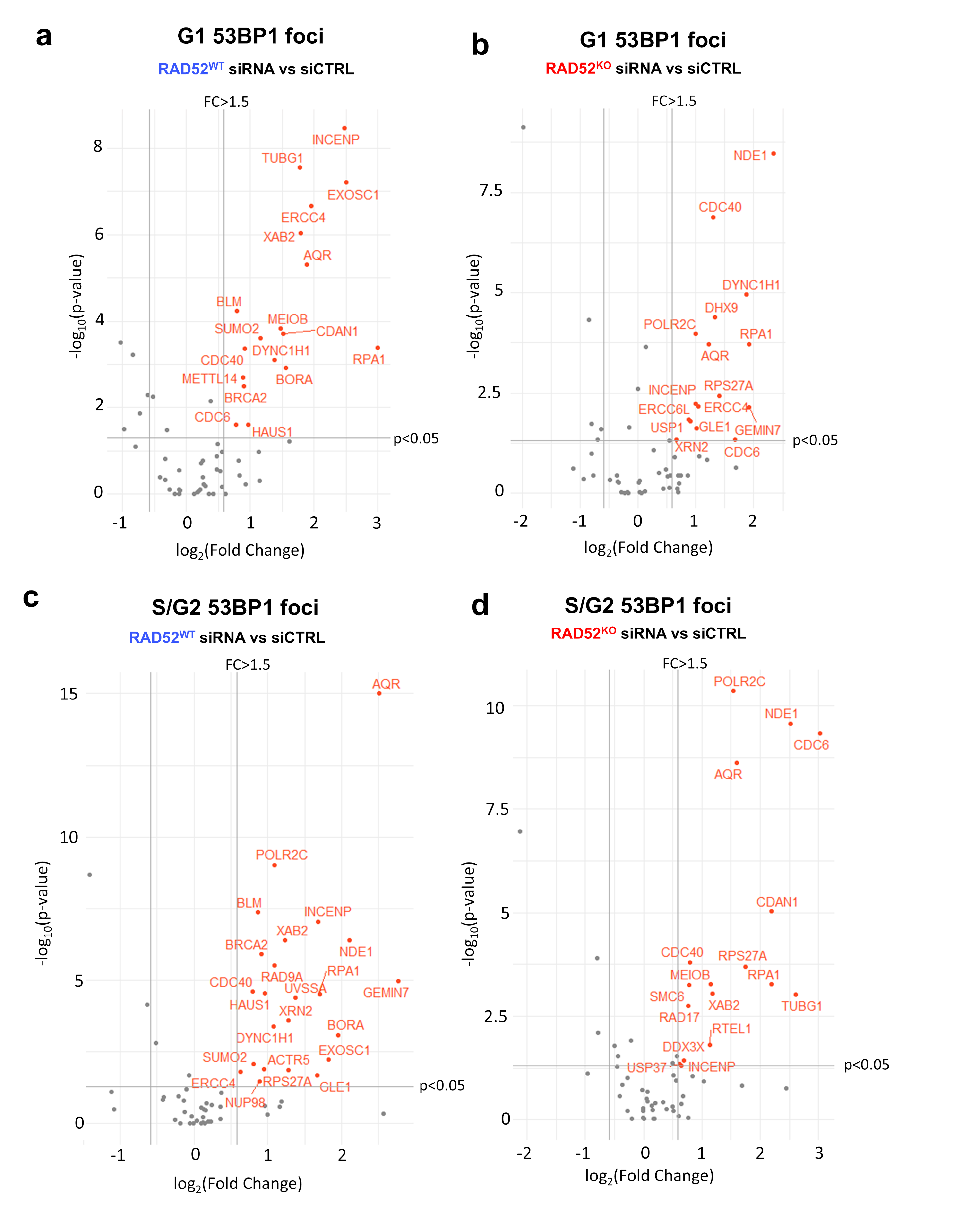

Supplement: S2 Fig — siRNA sub-screen hits from IN pathway groups 1–3 (59 genes + BRCA2, see Fig 1D) that have a significant (p<0.05 by Kolmogorov-Smirnov test) and >1.5-fold mean increase in G1 53BP1 foci in the RAD52WT cell line (a), G1 53BP1 foci in the RAD52KO cell line (b), S/G2 53BP1 foci in the RAD52WT cell line (c), and S/G2 53BP1 foci in the RAD52KO cell line (d) are shown in red. For all siRNAs shown, N>50 nuclei were analyzed in both RAD52KO and RAD52WT lines. All siRNAs used are pools of 4 siRNAs per gene. (TIF) [file pgen.1011479.s002.tif]

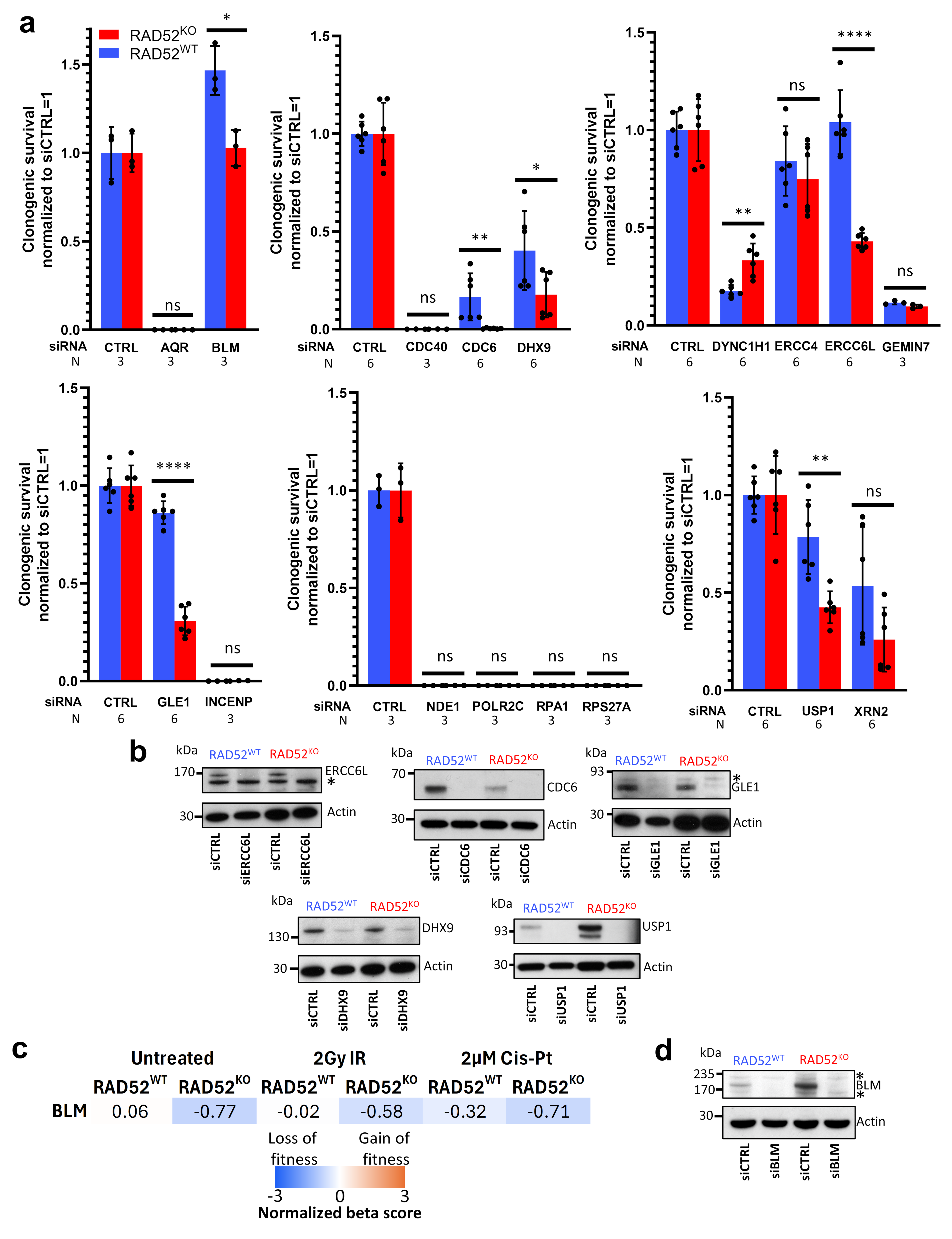

Supplement: S3 Fig — a) Clonogenic survival assay results for all 16 hits from the sub-screen (G1 53BP1 foci counts) and BLM. Colony count data is shown for RAD52KO vs RAD52WT lines with depletion of gene bys siRNA (pools of 4 siRNAs per gene) or siCTRL treatment and are normalized to respective siCTRL treated lines (siCTRL = 1). Statistical significance is determined by unpaired t-test, where ns = not significant, * = p<0.05, ** = p<0.01, *** = p<0.001, **** = p<0.0001. The number (N) of replicates is listed below each set of bars and reflects the number for both the RAD52KO and RAD52WT lines. b) Immunoblots confirming knock-down of genes that caused a significant viability defect in the RAD52KO cell line as compared to the RAD52WT line. Shown are ERCC6L, CDC6, GLE1, DHX9, and USP1 depletion via siERCC6L, siCDC6, siGLE1, siDHX9, and siUSP1 respectively in RAD52KO and RAD52WT cell lines. *non-specific band. c) Heatmap of normalized beta scores from the genome-wide screens (Fig 1B) for BLM. d) Immunoblots confirming knock-down of BLM by siBLM in the RAD52KO and RAD52WT cell lines. *non-specific bands. (TIF) [file pgen.1011479.s003.tif]

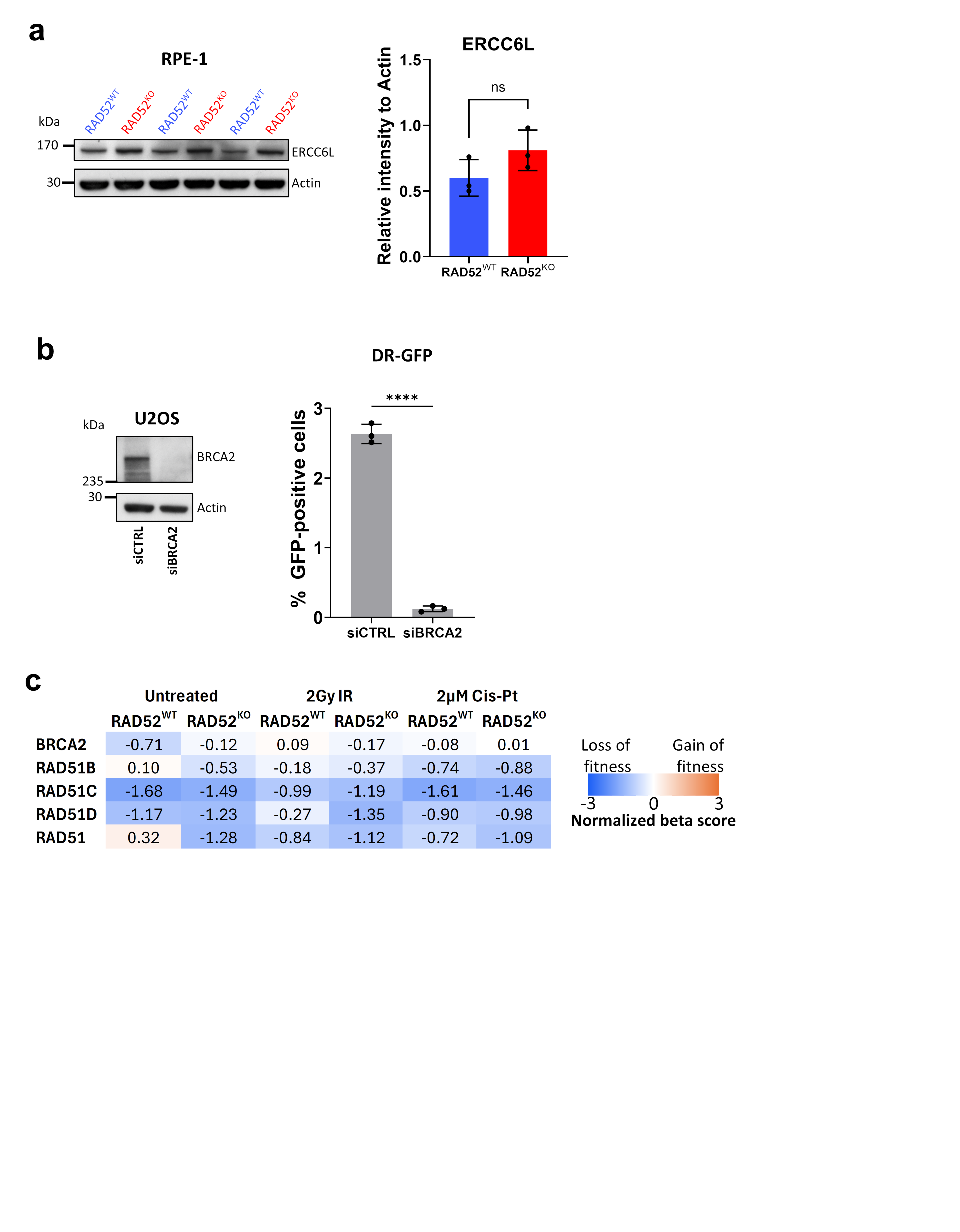

Supplement: S4 Fig — a) No significant difference in ERCC6L expression between RAD52KO and RAD52WT RPE-1 lines. (Left) Immunoblot analysis of RAD52KO and RAD52WT RPE-1 lines, each grown in 3 independent wells, to detect ERCC6L expression. (Right) Quantification of Immunoblot analysis. ns = not significant by t-test. b) Significant reduction in homology driven repair (HDR) efficiency after siBRCA2 treatment in U2OS cells. (Left) Immunoblot confirming BRCA2 depletion after siBRCA2 treatment in U2OS cells. (Right) Frequency of GFP-positive cells generated by the DR-GFP reporter (indicating repair by HDR) after siBRCA2 (pool of 4 siRNAs) or siCTRL treatment. N = 3 independent replicates. **** = p<0.0001 by t-test. c) Heatmap of normalized beta scores from the genome-wide screens (Fig 1B) for genes known to have synthetic lethal interactions with RAD52. (TIF) [file pgen.1011479.s004.tif]

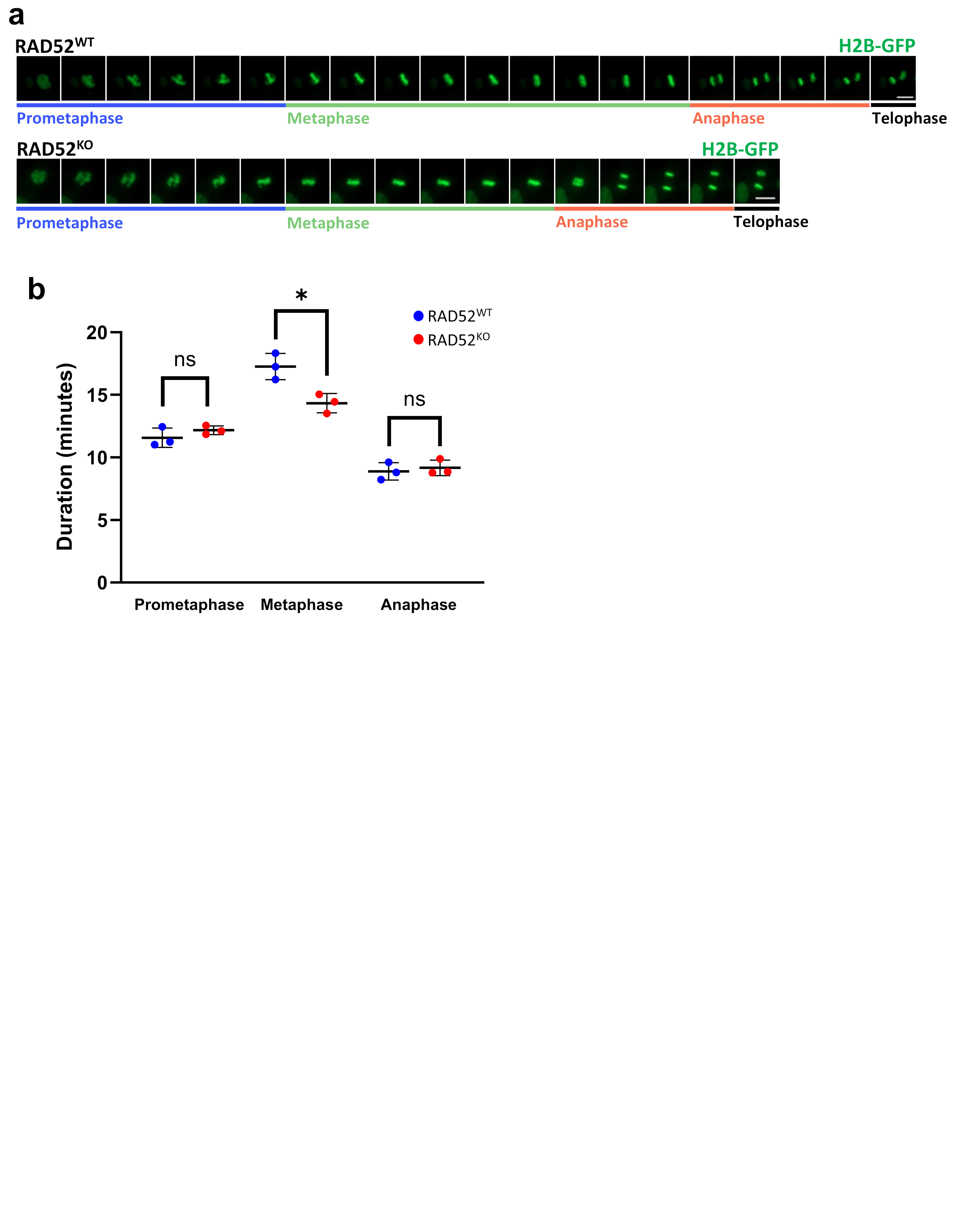

Supplement: S5 Fig — a) Representative examples of live cell imaging of mitotic cells in RAD52WT and RAD52KO cell lines. Stages of mitosis are defined below each example for the duration of the stage, except telophase (onset, identified by decondensation of chromatin). Cells were labeled with H2B-GFP and imaged at a rate of 2 min per frame. Scale bar is 20 μm, and images were taken at 20x magnification. b) Quantification of average phase duration for each cell line. Bars show average duration across N = 3 experiments, where >50 cells were analyzed from at least 2 fields of view over 12 hours per experiment. Significance determined by unpaired t-test. * = p<0.05, ns = not significant. (TIF) [file pgen.1011479.s005.tif]

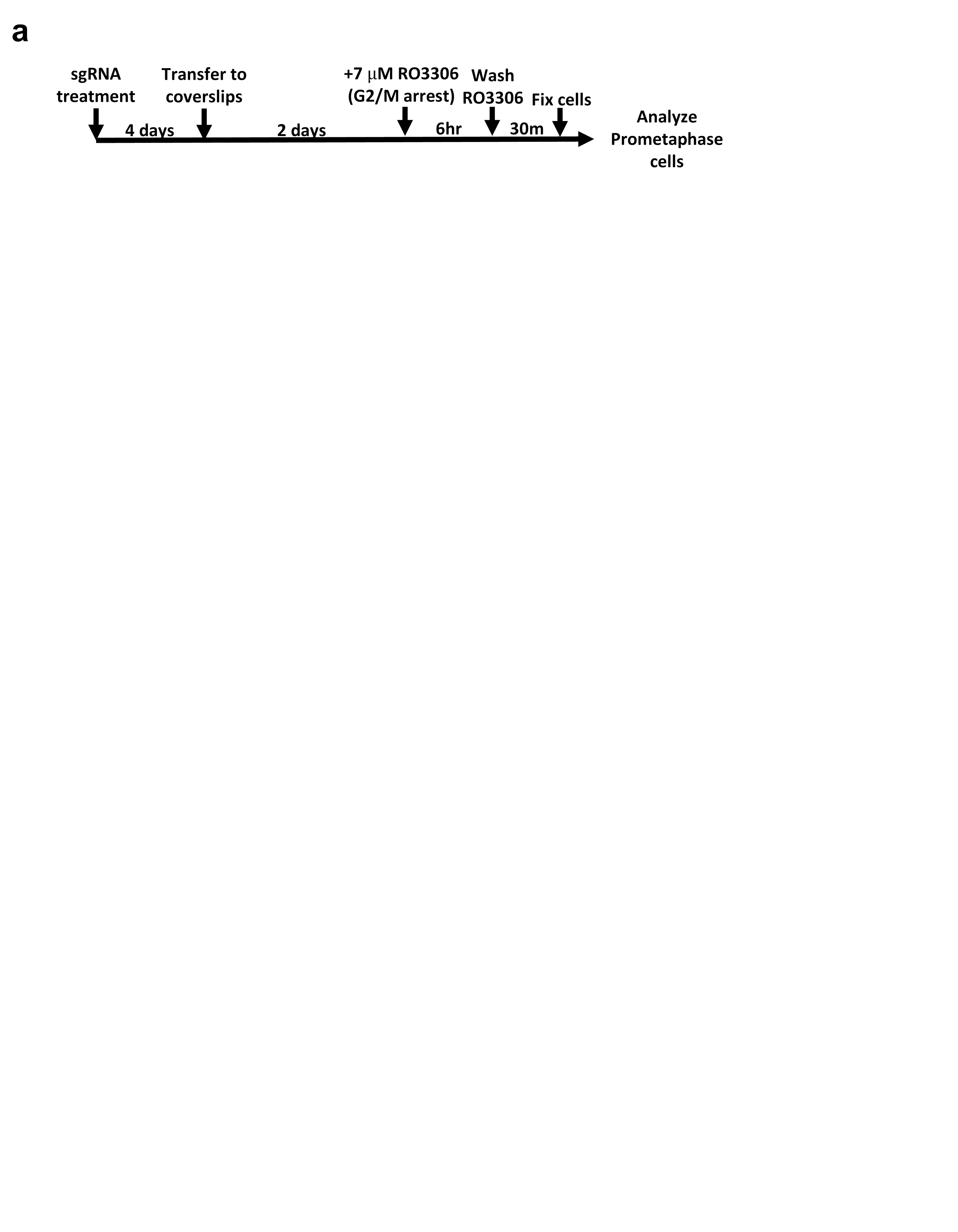

Supplement: S6 Fig — a) Schematic of treatment with sgRNAs (pool of 3) targeting ERCC6L used to examine RAD52-GFP foci in prometaphase cells. (TIF) [file pgen.1011479.s006.tif]

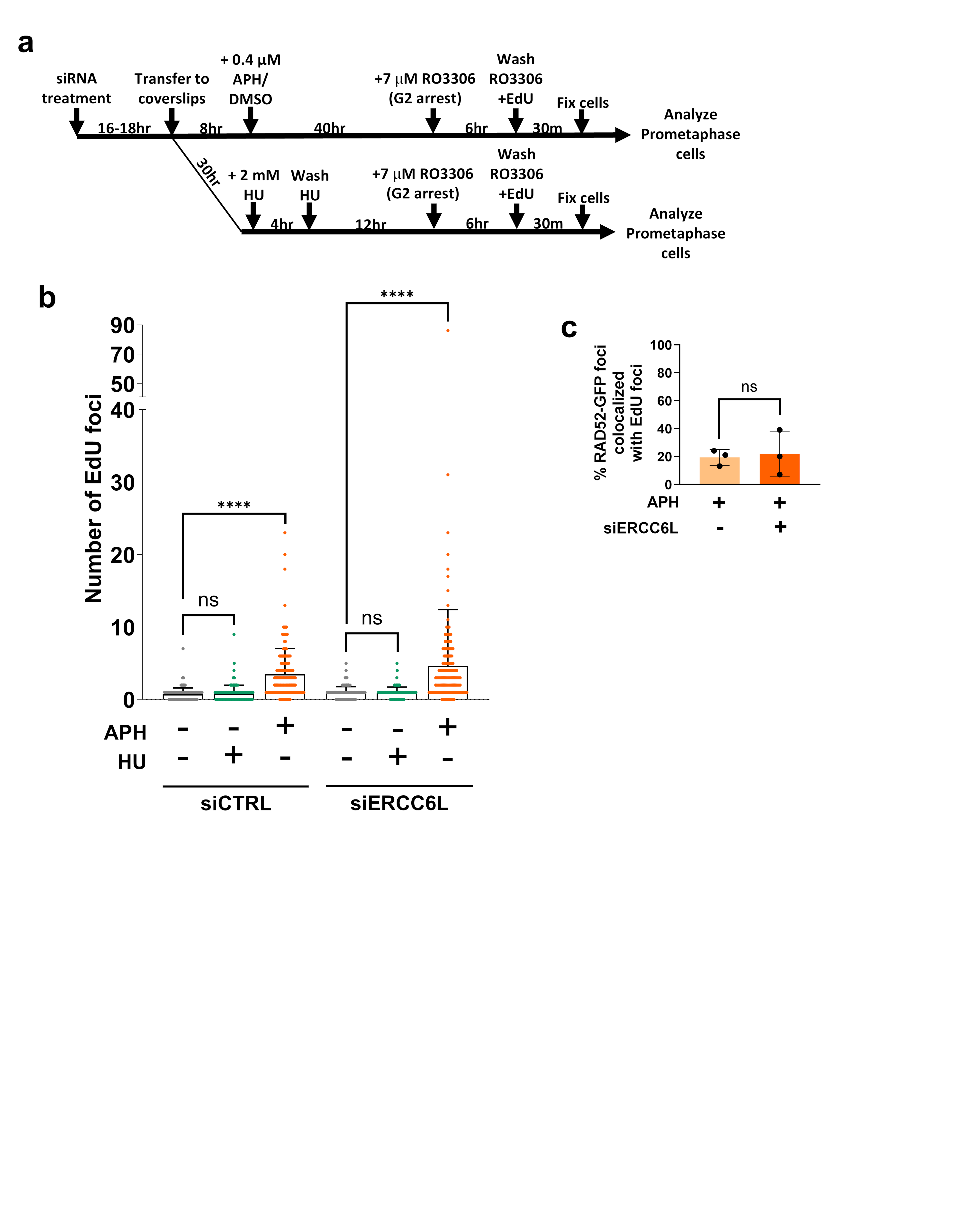

Supplement: S7 Fig — a) Schematic of treatments used for MiDAS detection in RAD52-GFP-expressing prometaphase cells exposed to either APH or HU. b) EdU foci increase upon APH exposure but not with HU exposure and recovery, irrespective of siERCC6L treatment (pool of 4 siRNAs). Bars show mean foci value. Significance determined by K-S test where ns = not significant and **** = p<0.0001. The number of nuclei (N) analyzed per condition are N = 157–162. c) The percentage of RAD52-GFP foci that colocalize with EdU foci in cells that have ≥5 EdU foci is not significantly different in cells treated with siCTRL or siERCC6L after APH exposure. Analysis of the data shown in (Fig 6C and 6D). N = 3 independent replicates. ns = not significant, unpaired t-test. (TIF) [file pgen.1011479.s007.tif]

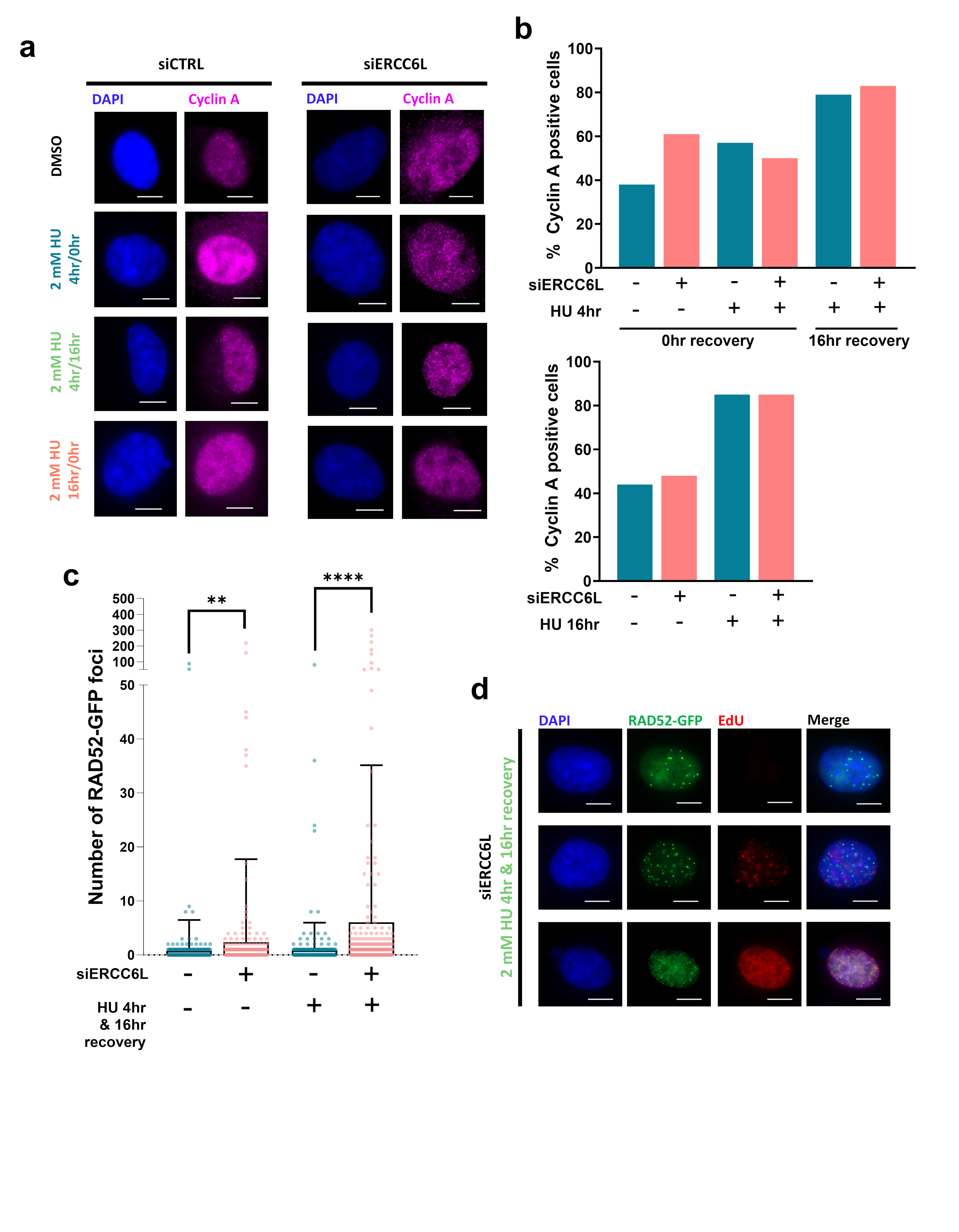

Supplement: S8 Fig — a) Example IF images of interphase cells with Cyclin A co-staining (RAD52 foci are shown in Fig 7B). Treatments with siERCC6L (pool of 4 siRNAs) or siCTRL and HU are as shown in Fig 7A. Scale bars are 10 μm and images were taken at 40x magnification. b) siERCC6L treatment does not substantially alter Cyclin A positive levels in interphase cells. HU treatment durations and recovery times are as shown in (Fig 7A, 7C and 7D). c) RAD52-GFP foci in interphase cells significantly increase with siERCC6L treatment (pool of 4 siRNAs) with and without HU treatment and recovery in experiments performed with EdU incorporation. Experiments performed as shown in (Fig 7E). Bars show mean foci value. The number of nuclei (N) analyzed per condition are N = 330–342. ns = not significant, *** = p<0.001, and **** = p<0.0001, K-S test. d) Representative images showing RAD52-GFP foci and EdU labeling in cells treated with siERCC6L and HU. Scale bars are 10 μm and images were taken at 40x magnification. (TIF) [file pgen.1011479.s008.tif]
